# A Plastic Biosynthetic Pathway for the Production of Structurally Distinct Microbial Sunscreens

**DOI:** 10.1021/acschembio.3c00112

**Published:** 2023-08-21

**Authors:** Sıla Arsın, Endrews Delbaje, Jouni Jokela, Matti Wahlsten, Zoë M. Farrar, Perttu Permi, David Fewer

**Affiliations:** † University of Helsinki, Department of Microbiology, Faculty of Agriculture and Forestry, 00014 Helsinki, Finland; ‡ University of São Paulo, Center for Nuclear Energy in Agriculture, Avenida Centenário 303, 13400-970 Piracicaba, São Paulo, Brazil; § Department of Chemistry, University of Jyväskylä, 40014 Jyväskylä, Finland; ∥ Department of Biological and Environmental Science, Nanoscience Center, University of Jyväskylä, 40014 Jyväskylä, Finland

## Abstract

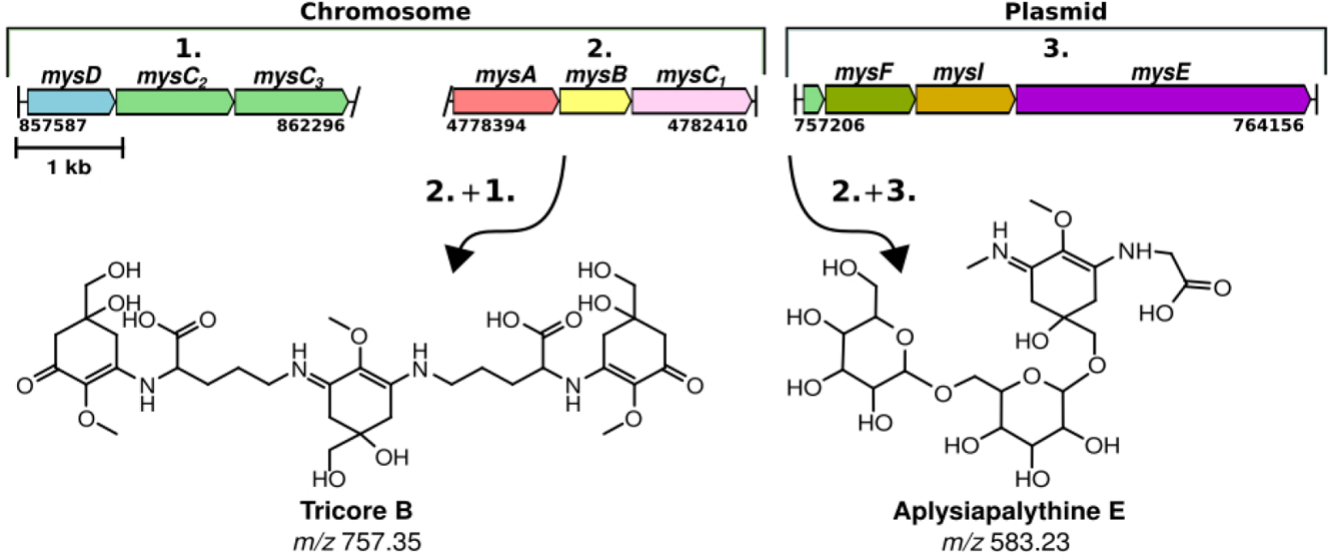

Mycosporine-like amino acids (MAAs) are small, colorless, and water-soluble secondary metabolites. They have high molar extinction coefficients and a unique UV radiation absorption mechanism that make them effective sunscreens. Here we report the discovery of two structurally distinct MAAs from the lichen symbiont strain *Nostoc* sp. UHCC 0926. We identified these MAAs as aplysiapalythine E (C_23_H_38_N_2_O_15_) and tricore B (C_34_H_53_N_4_O_15_) using a combination of high-resolution liquid chromatography–mass spectrometry (HR-LCMS) analysis and nuclear magnetic resonance (NMR) spectroscopy. We obtained a 8.3 Mb complete genome sequence of *Nostoc* sp. UHCC 0926 to gain insights into the genetic basis for the biosynthesis of these two structural distinct MAAs. We identified MAA biosynthetic genes encoded in three separate locations of the genome. The organization of biosynthetic enzymes in *Nostoc* sp. UHCC 0926 necessitates a branched biosynthetic pathway to produce two structurally distinct MAAs. We detected the presence of such discontiguous MAA biosynthetic gene clusters in 12% of the publicly available complete cyanobacterial genomes. Bioinformatic analysis of public MAA biosynthetic gene clusters suggests that they are subject to rapid evolutionary processes resulting in highly plastic biosynthetic pathways that are responsible for the chemical diversity in this family of microbial sunscreens.

## Introduction

Many organisms produce protective pigments and other secondary metabolites to protect themselves against the harmful effects of the ultraviolet radiation.^1^ Mycosporine-like amino acids (MAAs) are small, colorless, and water-soluble compounds that effectively absorb UV radiation between 310 and 360 nm with high molar extinction coefficients ranging between ε = 28,100 to 50,000 M cm^–1^.^2−4^ MAAs can dissipate 98% of the absorbed UV radiation as heat to the surroundings, and some variants such as mycosporine-2-glycine and glycosylated pophyra-334 derivatives possess considerable antioxidant activity.^5−7^ There is over three decades of interest in the potential application of MAAs in the pharmacological and cosmetic industries due to their effective UV absorbance, antioxidant activity and nontoxic nature.^8−11^

MAAs are secondary metabolites with over 70 chemical variants that consist of at least one cyclohexanone or cyclohexenimine chromophore where different amino acid substituents can be found bound to the first (C1) and the third (C3) carbon of the chromophore.^4^ Modification of the amino acid residues and/or the addition of sugar moieties further increase the structural diversity of MAAs.^4^ Shinorine (λ_max_ = 333 nm, 333 Da) and porphyra-334 (λ_max_ = 334 nm, 347 Da) are the most common MAA chemical variants reported.^12,13^ Shinorine and porphyra-334 biosynthesis proceeds through the action of four enzymes encoded in a compact biosynthetic gene cluster as either *mysABCD* or *mysABCE*.^14^ All known MAA biosynthetic enzymes are reported to be encoded in compact and coregulated biosynthetic gene clusters similar to other microbial secondary metabolites.^14−17^

The genus *Nostoc* is a particularly rich source of structurally diverse MAA chemical variants.^3,16,18,19^ Here we report the lichen-symbiont *Nostoc* sp. UHCC 0926 produces two distinct MAA chemical variants identified as tricore B (C_34_H_53_N_4_O_15_) and aplysiapalythine E (C_23_H_38_N_2_O_15_). Surprisingly, we identified three discontiguous MAA biosynthetic gene clusters encoded at distant locations in the complete genome of *Nostoc* sp. UHCC 0926. The organization of biosynthetic genes in *Nostoc* sp. UHCC 0926 necessitates a branched biosynthetic pathway to explain the biosynthesis of the two structurally distinct MAAs. We present a biosynthetic scheme to show how these distantly encoded enzymes might be working together, wherein we also propose the involvement of additional enzyme. Our bioinformatic analysis suggests that the MAA biosynthetic pathways of cyanobacteria can be highly plastic, explaining the chemical diversity observed for this family of microbial sunscreens.

## Results and Discussion

### Structural Characterization of Aplysiapalythine E and Tricore B

We detected two dominant MAA chemical variants (**18** and **19**), with peak absorption between 300 and 350 nm, by ultraperformance liquid chromatography with quadrupole time-of-flight (UPLC-QTOF) analysis of the 100% methanol extracts of the *Nostoc* sp. UHCC 0926 dried cell biomass (Table S1). We also detected and deduced the production of 20 additional MAA chemical variants according to their respective ion chromatograms (Figure S1) and MS^E^ spectra (Figures S2–S3). The most dominant MAA chemical variant (*m*/*z* of 583.23, **19**) accounted for 53% of the total peak area and possessed two hexose moieties (Table S1, Figure S1). The second major chemical variant (*m*/*z* = 757.35, **18**) comprised 18% of the total MAAs detected and contained three chromophores with two Ornithine (Orn) residues (Table S1, Figures S1 and S3).

**Figure 1:**
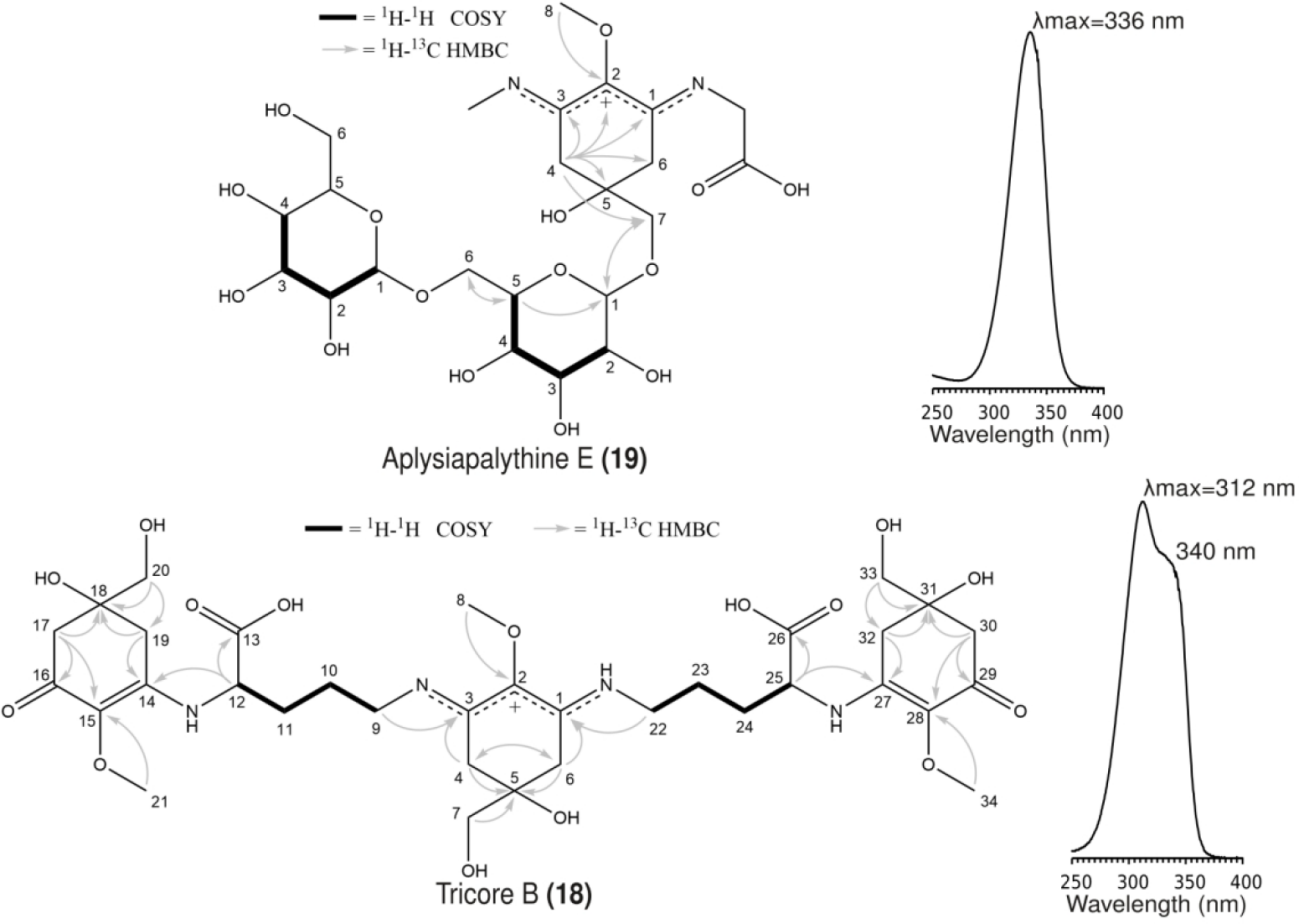
Chemical structures of the aplysiapalythine E (582 Da, λ_max_ = 336 nm) and tricore B (756 Da, λ_max_ = 312 nm) with a shoulder peak at 340 nm from the lichen symbiont *Nostoc* sp. UHCC 0926. ^1^H–^1^H COSY and ^1^H–^13^C HMBC correlations are given for each compound.

We isolated and dissolved the major MAA chemical variant (**19**) in D_2_O and ^1^H, ^13^C, ^1^H–^1^H DQF-COSY, ^1^H–^1^H TOCSY, ^1^H–^13^C HSQC-TOCSY, and edited ^1^H–^13^C HSQC and ^1^H–^13^C HMBC spectra were recorded (Table S2, Figures S4–S10). All δ_H_ and δ_C_ signals for atom positions 1–8 were typical for MAA chemical variants that contain a cyclohexenimine core unit (Table S2). Double signals, particularly from the CH_2_–4 core unit, indicated the presence of at least two cyclohexenimine core units in the sample with differing abundances (Table S2). Signals from two hexose sugars (Hex-1 and Hex-2) with relatively even abundances were recognized with almost identical anomeric signals (CH-1) but without clear recognition of CH-5 and CH-6 signals (Table S2). HMBC correlation from the first hexose (Hex-1) anomeric proton 1 to MycA carbon 7 seemed to be present, and accordingly, from MycA-7a to Hex1–1 suggested that Hex1 was connected to the 7-OH (Figure 1). Assuming that Hex1 CH-6 was correctly annotated to be δ_H_ 3.88 and 4.04 ppm and δ_C_ 69.1 ppm, then the second hexose (Hex2) was probably connected to Hex1 to position 6 (Figure 1). Both hexopyranoses (Hex1/2) have a coupling constant ^3^*J*_H1,H2_ of 7.8 Hz meaning that both H1 and H2 are axial, which is the case for β-d-glucose and β-d-galactose.^20^ Proton spectrum from hydrolyzed MAA (**19**) demonstrated the presence of both glucose and galactose (Figure S11). NMR data obtained did not allow us to determine the position of the hexose units. There was also a low intensity doublet signal δ_H_ 5.24 (3.9 Hz) and δ_C_ 92.1, which best matched with free α-d-glucose in water solutions in 2:3 equilibrium with the β-anomer (Figure S11). The product ion spectrum of the protonated MAA (**19**) showed the loss of two hexose units and that the aglyconic part matched structurally best to aplysiapalythine C (Figure S12 and Table S3). The mass spectrometric data best fitted to glycosylated aplysiapalythine C structure. However, NMR analysis could not verify the presence of either methyl or −CH_2_COOH groups in the NH positions of the cyclohexenimine MAA core structure. A UV maximum at 336 nm of 582 Da MAA supports aplysiapalythine-C-like structure as cyclohexenimine-type MAA core unit with ligands in both N atoms have reported maxima between 325 and 362 nm and with one ligand between 320 and 322 nm (Figure 1).^7^ The extinction coefficient of this variant was calculated as ε = 12,390 L mol^–1^ cm^–1^ at 336 nm. We named the novel MAA (**19**) chemical variant aplysiapalythine E, and to the best of our knowledge, this is the first diglycosylated aplysiapalythine variant reported.^21−23^

The second major MAA chemical variant (**18**) had a major UV absorption maximum (λ_max_) at 312 nm, which fits to the two cyclohexenone chromophores in the 756 Da structure, and a shoulder at 340 nm, which fits to one cyclohexenimine with two N-ligands in the 756 Da structure (Figure 1). We isolated and dissolved the second major MAA chemical variant (**18**) in D_2_O and collected ^1^H, ^13^C, ^1^H–^1^H-COSY, ^1^H–^1^H-TOCSY, ^1^H–^13^C-HSQC, and ^1^H–^13^C-HMBC spectra to resolve the structure of this chemical variant (Table S4, Figures S13–S18). All chemical shift values (δ_H_ Δ ± 0.03 ppm and δ_C_ Δ ± 0.4 ppm) and the COSY, TOCSY, HSQC, and HMBC correlations were in full agreement with the previously characterized 756 Da MAA, reported from *Nostoc flagelliforme* CCNUN1.^16^ We also calculated the estimate extinction coefficient for this variant at 312 nm as ε = 58,970 L mol^–1^cm^–1^. The 1050 Da glycosylated MAA isolated from *Nostoc commune* was the first characterized MAA chemical variant that was composed of three chromophores.^18,24^ To establish a more practical naming convention, we choose to refer to the 1050 Da MAA as tricore A^18,24^ and the 756 Da aglycon variant discovered later,^16^ and reported here, as tricore B.

### Branched Biosynthetic Pathway for the Synthesis of Tricore B and Aplysiapalythine E

**Figure 2:**
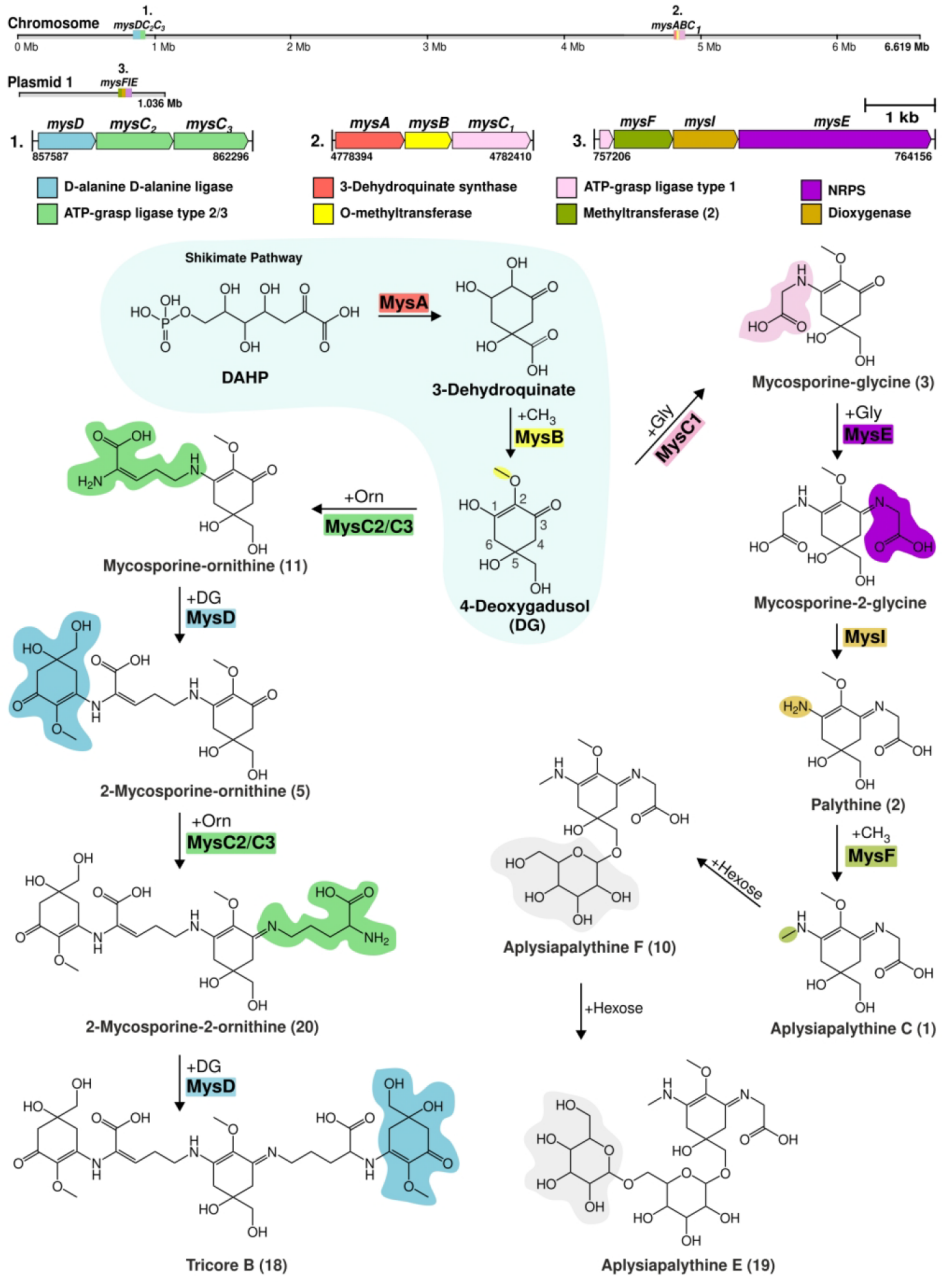
Proposed branched biosynthetic pathway for the synthesis of tricore B and aplysiapalythine E variants identified from *Nostoc* sp. UHCC 0926. The intermediate structures are numbered according to HR-LCMS data (Table S1). 4-Deoxygadusol synthesis is highlighted as it a shared biosynthetic intermediate for the biosynthesis of both the tricore B and aplysiapalythine E.

Tricore B and aplysiapalythine E are structurally distinct from one another, which suggested that the genome of *Nostoc* sp. UHCC 0926 encodes two different biosynthetic pathways. We sequenced the genome of *Nostoc* sp. UHCC 0926 using PacBio technology, and the resulting complete assembly length was 8,314,159 bp in total, comprising a single circular chromosome and six plasmids (Table S5). Interestingly, genes encoding MAA biosynthetic enzymes were identified at three different locations on the genome with two biosynthetic gene clusters encoded on the chromosome and one such biosynthetic gene cluster on a plasmid (Figure 2). A 4.7-kb biosynthetic gene cluster encoded MysD, MysC_2_, and MysC_3_ (Figure 2), while a 8.2-kb biosynthetic gene cluster encodes MysA, MysB, and MysC_1_ (Figure 2). A third 6.9-kb biosynthetic gene cluster encoded MysF, MysI, and MyseE and was located on the largest plasmid (Figure 2, Table S6). MysF contains a methyltransferase domain, while MysI is a TauD/TfdA family dioxygenase, distinct from the previously described MysH enzyme,^17^ and finally the MysE is an NRPS enzyme (Table S6). We also identified and annotated a 243-bp pseudo *mysC*_*1*_ gene with a premature stop codon located upstream of the *mysFIE* cluster (Figure 2, Table S6).

We propose a biosynthetic scheme to explain the production of the structurally distinct tricore B and aplysiapalythine E in *Nostoc* sp. UHCC 0926 was based on structural intermediates detected as well as established biochemical reactions and bioinformatic predictions (Figure 2, Tables S1,S6). MysA is a phosphate cyclase that acts on either sedoheptulose-7-phosphate from the pentose phosphate pathway or the 3-deoxy-d-arabino-heptulosonate-7-phosphate (DAHP) from the shikimate pathway to produce either dimethyl-4-deoxygadusol or 3-dehydroquinate, respectively.^14,25−28^ MysB is an O-methyltransferase, which methylates a 3-dehydroquinate intermediate to form the 4-deoxygadusol chromophore.^14^ The MysC is an ATP-grasp ligase that catalyzes the addition of glycine (Gly) onto the C1 forming mycosporine-glycine.^14^ These first three steps in the biosynthesis of MAAs have been characterized through biochemical assays and heterologous expression experiments.^14^ Tricore B biosynthesis most likely proceeds with the addition of an Orn residue onto the first carbon C1 of the 4-deoxygadusol either by the MysC_2_ or MysC_3_ enzymes as previously proposed.^14,16^ The MysC_2_ or MysC_3_ enzymes are the new members of the MysC family that have yet to be characterized in biochemical assays (Figure 2).^16,29^ We detected the presence of the expected mycosporine-ornithine intermediate (**11**) in the extracts of *Nostoc* sp. UHCC 0926 (Figure 2, Table S1). The MysD, a d-alanine d-alanine ligase, is reported to catalyze the addition of Ser onto the C3 in shinorine biosynthesis.^14^ MysD can have high substrate affinities to either Ser or Thr while also showing a degree of promiscuity.^17^ We propose that MysD catalyzes the linkage between the Orn residue and C1 of another 4-deoxygadusol to form the 2-mycosporine-ornithine (**5**) (Figure 2).^14,16^ The second Orn residue would then be attached onto the C3 of the core 4-deoxygadusol by MysC_2_/MysC_3_ to form the 2-mycosporine-2-ornithine intermediate (**20**) (Figure 2).^16^ Finally, tricore B (**18**) biosynthesis is likely completed with the attachment of the final Orn residue on to the third 4-deoxygadusol core by the MysD enzyme (Figure 2).

The biosynthesis of aplysiapalythine E starts with the synthesis of mycosporine-glycine (**3**), which is formed with a Gly residue addition onto the C1 of the 4-deoxygadusol through the successive action of MysA, MysB, and MysC (Figure 2). This reaction sequence has exact precedence in the biosynthesis of shinorine and porphyra-334.^14,17^ We propose that the MysE enzyme, a NRPS protein, catalyzes the linkage of a second Gly residue onto the C3 of mycosporine-glycine forming mycosporine-2-glycine (Figures 2, 4). The MysE protein is reported to catalyze the addition of Ser to mycosporine-glycine (**3**) in the biosynthesis of shinorine.^14^ However, we could not detect mycosporine-glycine (**3**) by HR-LCMS (Figures 2, 4). We propose that the Gly residue bound on C3 is hydroxylated and destabilized by MysI, leading to the spontaneous formation of a palythine (**2**) intermediate (Figure 2). This proposed reaction is similar to the proposed mechanism of MysH.^17^ Recently, a phytanoyl-CoA dioxygenase enzyme, now named MysH, was reported to play a central role in the biosynthesis of palythine (λ_max_ = 320 nm, 244 Da) variants.^17^ MysH is predicted to hydroxylate Gly on C1 and destabilize the side chain leaving an amide residue, resulting in palythines.^17^ However, MysI would act on a Gly residue on C3 rather than C1 in our putative biosynthetic scheme (Figure 2). This reaction mechanism requires further experimental validation. We predict that the MysF enzyme methylates the amide residue attached to C3 of the 4-deoxygadusol forming the aplysiapalythine C (**1**) intermediate (Figure 2). Finally, the aplysiapalythine E (**19**) variant is formed by the attachment of a hexose sugar onto the C7 of aplysiapalythine C to form aplysiapalythine F (**10**) and then another hexose onto C6 of the first attached hexose sugar (Figure 2). The glycosyltransferase enzymes involved in these reactions are currently unknown and are likely to be encoded elsewhere in the genome, as previously reported for all glycosylated MAAs.^30,31^ It is possible that the glycosylation of MAAs is an additional process solely for the purpose of integration into the extracellular matrix and are regulated by other factors such as desiccation stress.^32^

### Plasticity of MAA Biosynthetic Pathways in Cyanobacteria

**Figure 3:**
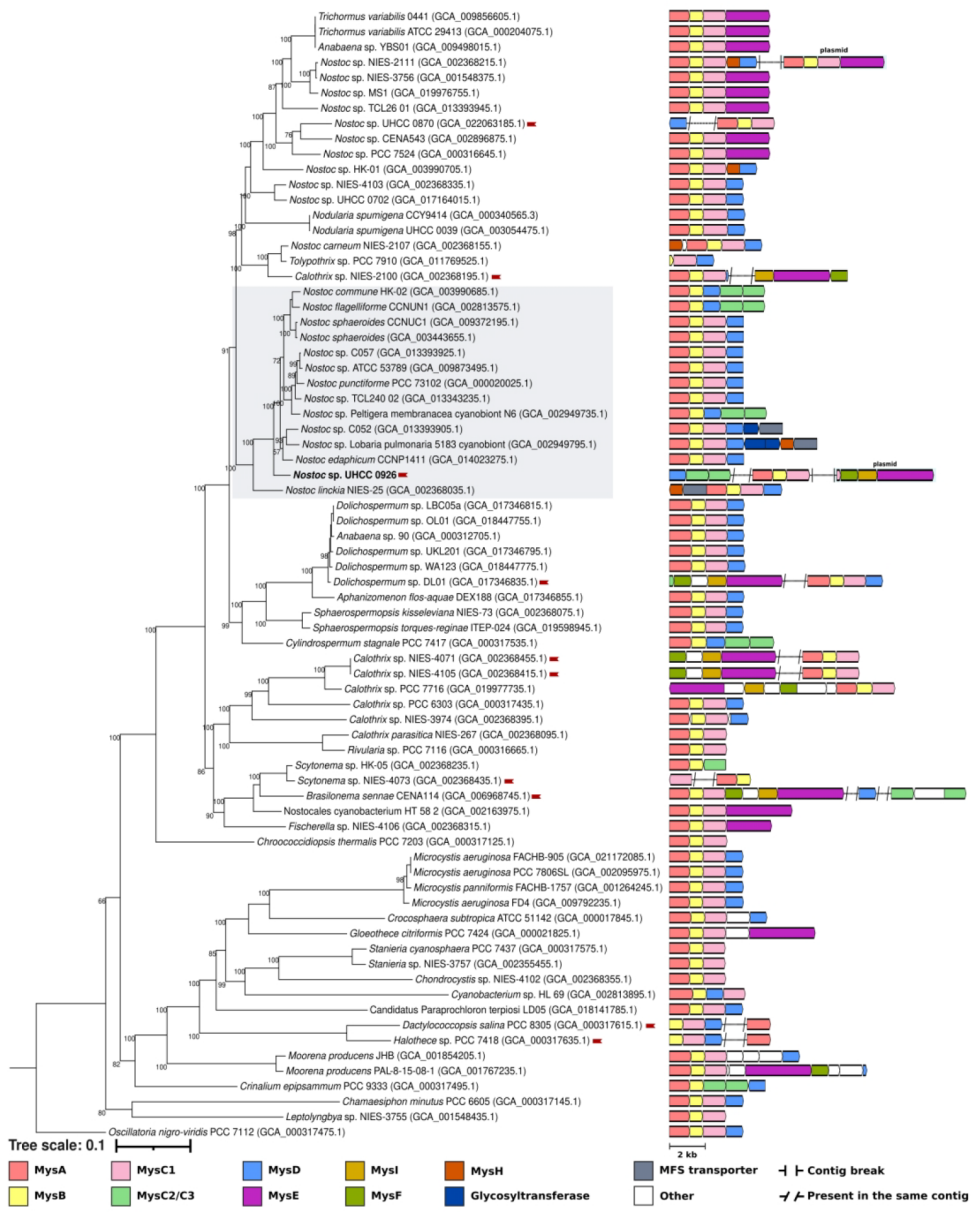
A maximum-likelihood phylogenomic tree of the complete cyanobacterial genomes that possess a MAA biosynthetic gene cluster. Tree was constructed using the sequences of 120 bacterial single-copy marker proteins with PROTGAMMAIGTR model with 1000 bootstraps. Cyanobacteria strains with discontiguous MAA biosynthetic gene clusters are marked with a red ribbon.

We extended our analysis to 293 high quality publicly available complete genomes to determine whether discontiguous MAA pathways are a common occurrence in cyanobacteria. We identified 75 cyanobacteria that possessed a type of a MAA biosynthetic gene cluster and constructed a phylogenomic tree to investigate their evolutionary distribution (Figure 3). We identified MysF, a methyltransferase that is distinct from MysB, encoded in 8 of the MAA biosynthetic gene clusters we analyzed (Figure 3, Table S6). We also identified two distinct dioxyganases, MysH and MysI, encoded in MAA biosynthetic gene clusters (Figure 3).

The overall distribution of MAA biosynthetic gene clusters lacks an obvious pattern in cyanobacteria (Figure 3). However, we detected 9 cyanobacteria with similar discontiguous MAA biosynthetic pathways (Figure 3). The sizes of these discontiguous MAA gene clusters ranged from 2.7 to 7.4 bp and encoded 1–4 proteins (Figure 3). Interestingly, *Nostoc* sp. NIES 2111 encodes two complete MAA biosynthetic gene clusters where *mysABCE* is on the chromosome and *mysABCD* is located on the plasmid (Figure 3). Interestingly, apart from the *Nostoc* sp. UHCC 0926, this is the only other strain to carry an MAA biosynthetic gene cluster on a plasmid as well as the chromosome (Figure 3). Our analysis also included *Tolypothrix* sp. PCC 7910 with an MAA biosynthetic pathway only composed of *mysCD* and a truncated pseudo *mysB* gene (Figure 2). Truncated genes were also identified in the *Nostoc* sp. UHCC 0926 (*mysC*_*1*_), *Dolicosphermum* sp. DL01 (*mysC*_*2*_), *Moorena producens* PAL-8-15-08-1 (*mysD*), and *Calothrix* sp. NIES-2100 (*mysD*). The presence of discontiguous and/or multiple MAA biosynthetic gene clusters together with pseudogenes is an indication that MAA biosynthetic pathways are undergoing constant evolutionary processes. Serial horizontal gene transfer (HGT) events might be the main way MAA biosynthetic pathways obtain new biosynthetic genes: for example, through the independent acquisition and gradual decay of two or more MAA biosynthetic gene clusters, which later merge to form novel MAA biosynthetic gene clusters.

Phylogenetic analysis of individual MAA biosynthetic enzymes allowed us to identify and confirm the distantly located MAA biosynthetic enzymes, as well as to investigate their evolutionary distribution (Figures S19–S23). Individual phylogenetic distribution of MysA, MysB, and MysC_1_ biosynthetic enzymes appear to be in line with cyanobacterial taxonomy (Figures S19–S21) especially among the genus *Nostoc*.^33^ This finding is consistent with previous literature as MAA core forming MysA, MysB, and MysC enzymes are thought to be conserved in all MAA producing organisms.^34,35^ The formation of two distant clades in the MysA phylogenetic tree could be linked to difference between 2-epi-5-epi-valiolone synthase and 3-dehydroquinate synthase-type MysA enzymes (Figure S19).^28^ Additionally, the topology of the phylogenetic tree constructed using methyltransferase enzymes further indicated that the MysF enzymes found in the MAA biosynthetic gene clusters are indeed distinct from MysB enzymes as they formed a distant clade of their own (Figure S20).

We constructed a phylogenetic tree based on the MysC protein (Figure S21). The Gly-specific MysC_1_ is conserved, and the Orn/Lys specific MysC_2_ and MysC_3_ enzymes form their own clade as likely more recent derivatives of the MysC_1_ (Figure S21). As expected, the MysC_2_ and MysC_3_ of *Nostoc* sp. UHCC 0926 is found in the same clades as other known tricore MAA forming cyanobacteria including *Nostoc flagelliforme* CCNUN1 and *Nostoc commune* HK-02 (Figure S21). Additionally, all the cyanobacteria in these highlighted clades possess the *mysDC*_*2*_*C*_*3*_ cluster formation, which is also consistent for the phylogenetic tree constructed for MysD enzyme (Figures S21 and S22), where the clade in which MysD of *Nostoc* sp. UHCC 0926 places along with other cyanobacteria with the *mysDC*_*2*_*C*_*3*_ cluster organization (Figure 3). This particular gene cluster organization was associated with tricore MAA synthesis and are specifically common in draft resistant cyanobacteria as a likely form of habitat adaptation.^29^

**Figure 4:**
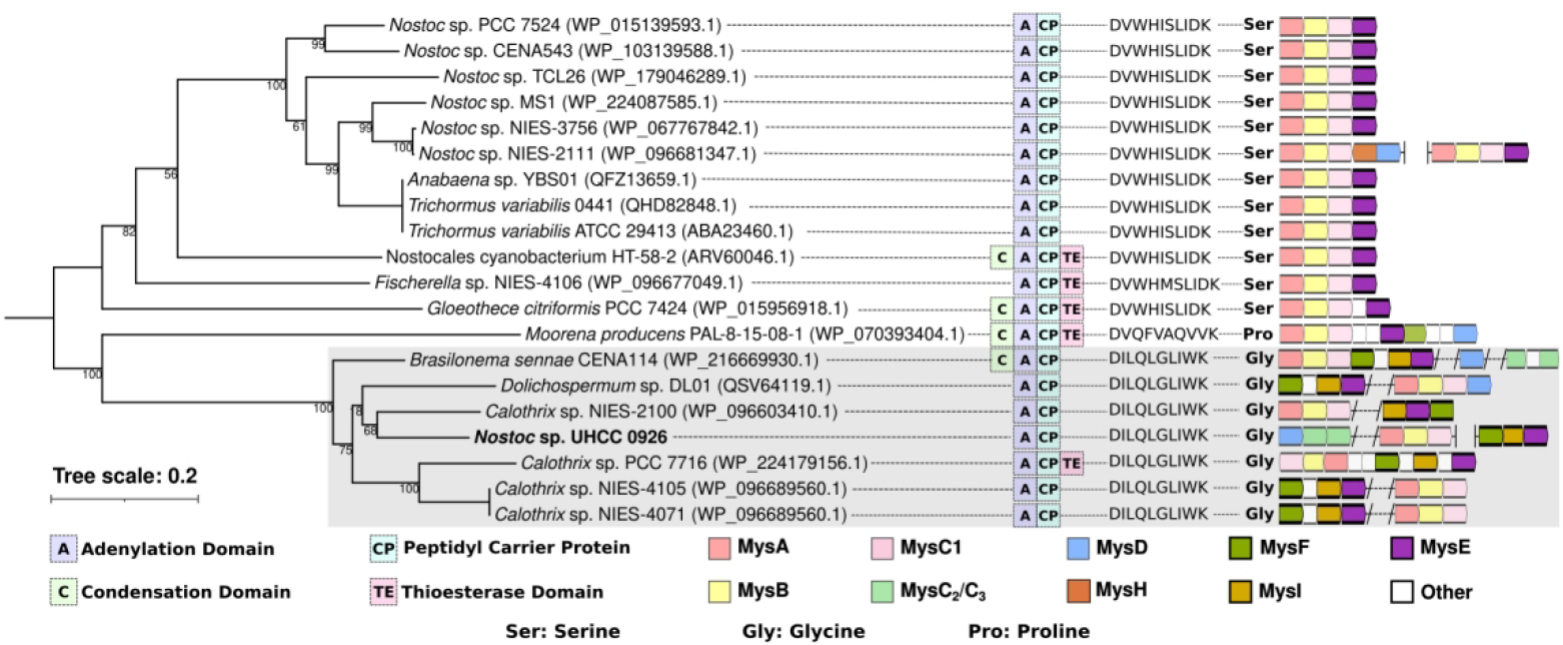
Distribution, domain architecture, and substrate variation including the Stachelhaus codes of the MysE enzymes found in MAA biosynthetic gene clusters of the analyzed cyanobacterial genomes. MysE enzymes are colored brighter alongside the MysF (dark green) and MysI (mustard yellow) to highlight the coclustering pattern.

We investigated the distribution of the MysE enzymes together with their respective enzymatic properties (Figure 4). Interestingly, the phylogenetic distribution of the MysE enzymes is congruent with the corresponding substrate specificities (Figure 4). The topology of the phylogenetic tree consists of two main clades where the first clade is composed of 12 Ser-specific MysE enzymes (Figure 4). Among these, the most common domain architecture is A-CP apart from *Gloeothece citriformis* PCC 7424 and *Nostocales cyanobacterium* HT-58-2 with C-A-CP-TE and *Fischerella* sp. NIES-4106 with A-CP-TE (Figure 4). The second main clade is composed of eight members, including the *Nostoc* sp. UHCC 0926, which forms a subclade with the six others. The subclade members are all specific for Gly, also sharing the domain architecture of A-CP apart from the *Calothrix* sp. PCC 7716 with A-CP-TE (Figure 4). MysE of *Moorena producens* PAL-8-15-08-1 places outside of this subclade as the only proline (Pro) specific MysE with the domain structure of C-A-CP-TE. Previously MysE activity seemed to be specific to Ser and shinorine synthesis.^14,31^ Our work demonstrates that there is a cryptic diversity among the MysE enzymes, where there are at least three MysE derivatives varying in their substrate specificities, which can be Ser, Gly, or Pro (Figure 4).

The phylogenetic tree constructed using the dioxygenase protein sequences places the MysH of *Nostoc linkia* NIES-25 in a clade with four others that belong to *Nostoc* sp., and the TauD/TfdA family of dioxygenases of *Nostoc* sp. UHCC 0926 is distantly in another clade with six others (Figure S23). The clear division suggested that there are two different types of dioxygenases involved in MAA biosynthetic pathways, and thus, we named the TauD/TfdA family dioxygenases as MysI (Figure S23). We propose that MysI is specific for Gly bound to C3 while MysH acts on Gly bound to C1 (Figure 2).^17^ These two enzymes are likely to have been recruited to the MAA biosynthetic pathways independently, with the palythine structure having arisen through convergent evolution on at least two occasions (Figure S23).

We have also noted a pattern emerging with the three new enzyme variants, MysF, MysI, and the Gly-specific MysE were consistently found adjacent to each other in all MAA biosynthetic gene clusters in which they were present (Figure 4). Interestingly, all of these biosynthetic gene clusters are also discontiguous, including the *mysFIE* in *Nostoc* sp. UHCC 0926, which is encoded on a plasmid (Figures 3, 4). The *mysFIE* biosynthetic gene cluster could be a remnant of a functional MAA biosynthetic gene cluster obtained via a HGT event and is slowly being integrated into the pre-existing biosynthetic gene cluster with the ancestral MAA enzymes of the cyanobacteria (Figures 2, 3).

*Nostoc* sp. UHCC 0926 seems to be a rare case because this strain encodes three distinct MAA biosynthetic gene clusters, the expression of which needs to be coordinated for the synthesis of the two main MAA variants (Figures 2, 3). Discontiguous biosynthetic gene clusters and branched biosynthesis of MAAs have not been previously reported. However, the phenomenon is not entirely unprecedented as there are examples of other secondary metabolites pathways that require coordinated expression of distant genes.^36,37^ MAA biosynthetic pathways likely date far back and had diversified through several HGT events, accumulation of mutations, gene duplications, deletions, and recombination events.^15,37,38^ As UV radiation is a constant threat, the evolutionary pressure to produce the most useful MAA chemical variants is ever-present.^39,40^ Here, the three distant MAA biosynthetic gene clusters work together in *Nostoc* sp. UHCC 0926 could be considered a snapshot of dynamic evolutionary processes hard at work.

## Conclusions

Here we report the identification of three distantly located MAA biosynthetic gene clusters from the *Nostoc* sp. UHCC 0926 genome, which may be acting in tandem to synthesize tricore B and aplysiapalythine E via a branched biosynthetic pathway. Our extended analysis of the similarly organized MAA biosynthetic gene clusters in publicly available complete cyanobacterial genomes provides valuable insight into the evolutionary progression and the subsequent plasticity of the MAA biosynthetic pathways. Additionally, we predicted roles for new types of methyltransferase and dioxygenase enzymes which might be involved in the synthesis of aplysiapalythines. Overall, our data shows that the MAA biosynthetic pathways can be much more diverse and complex than previously assumed.

## Materials and Methods

### *Nostoc* sp. UHCC 0926 Biomass Cultivation and Harvest

*Nostoc* sp. UHCC 0926 is a lichen symbiont, isolated from central Finland. The non-axenic culture is maintained in 40 mL modified Z8 media^41^ lacking a source of combined nitrogen at 20 °C and under the light intensity of 8.70 μmol m^–1^ s^–1^. For the purification of MAAs, we grew *Nostoc* sp. UHCC 0926 in large batches of 40 L for 4 weeks. 10 g dried cell biomass was obtained by harvesting the cultures via centrifugation using the Sorvall Lynx 6000 (Thermo Scientific) at 9000 g for 8 min at 20 °C and lyophilization with the Christ LCS Plus Beta 2–8 LCS Plus Freeze-Dryer at 0.0650 mbar for 48 h.

### Detection of MAAs by LC-MS-UV

Approximately 50–100 mg of dried cell biomass was collected in 2 mL plastic screw-cap Eppendorf tubes filled with 200 μL of 0.55 mm Glass Micro Beads (Scientific Industries) were added to a screw-top Eppendorf tube. Cells were then disrupted in 1 mL of 100% methanol at 6.5 m s^–1^ for 20 s using a Fast Prep-24 (MP Biomedicals). Once lysed, the samples were centrifuged for 5 min at 10,000 g using an Eppendorf centrifuge 5415D. 100 μL of the supernatant was taken up and filtered using an Injekt-F 1 mL syringe (B-Braun) with 0.2 μm Fisherbrand PTFE syringe filter tip (Fisher Scientific) into a short thread sample vial (VWR) for LC-MS analysis.

An initial method of screening was performed using UPLC-QTOF (Acquity I-Class UPLC-SynaptG2-Si, Waters Corp., Milford, MA, USA) with the ACQUITY UPLC BEH Amide Column, 130 Å, 1.7 μm, 2.1 mm × 100 mm (Waters Corp., Milford, MA) with solvent A: 0.2% ammonium formate and solvent B: acetonitrile with a flow rate of 0.300 mL min^–1^. The initial percentages of solvents were 10% solvent A and 90% solvent B, which changed linearly to 40% solvent A and 60% solvent B by 9.00 min. The sample was injected 0.5 μL at a time. The target sample temperature was 5.0 °C and column temperature: 40.0 °C. Samples were run at ES+ polarity with the capillary voltage at 2.5 kV. The sampling cone was set to 20 V with a source temperature of 120 °C and desolvation temperature of 600 °C. The cone gas flow was set to 50 L h^–1^ and desolvation gas flow to 1000.0 L h^–1^ with a nebulizer gas flow of 6.0 bar. Photodiode array detector recorded between 210 and 800 nm.

### Purification of MAAs

10 g of *Nostoc* sp. UHCC 0926 dried biomass was processed 1 g at a time in 30 mL of 100% methanol, starting with cell breaking using the SilentCrusher M (Heidolph Instruments), at up to 20,000 rpm for 50 s. Samples were then centrifuged at 5000 g for 10 min at 10 °C, and the supernatants were collected into a 500 mL Rotary Evaporator Flask containing 10 mL of the ODS Chromatorex silica beads (Fuji-Davison Chemical Ltd., Aichi, Japan). Silica bound extract was then dried using a Büchi Rotavapor R-200. Phenomenex SPE strata SI-1 silica 5 g/20 mL cartridge columns were used to separate MAAs from other other pigments and compounds. Columns were primed with 20 mL of dichloromethanol (DCM) and then 10 mL of heptane. The flow of the solvents was aided by air pressure applied from the top. We then loaded 1 g of the silica bound dry cell extract onto the column and fractionated with a series of solvents, starting with 10 mL of heptane, followed by ethyl acetate, DCM, acetone, and 20 mL of methanol, all collected into 20 mL glass tubes. MAAs were then detected in the final methanol fractions using an Oridoe UV-1800 (Shimadzu) spectrophotometer by detection of peak absorbance at 330 nm.

We removed methanol using Turb Vap LV Evaporator at 28 °C and dissolved the sample in 1 mL of ultrapure water. We homogenized the sample by vortexing at full speed for a minute and sonicating with Sonorex Super 10P (Bandelin) bath sonicator for 5 min. Sample was then centrifuged for 2 min at 14,000 g using an Eppendorf Centrifuge 5415D F45-24-11. The 10 μL of the clear supernatant containing MAAs was analyzed with reverse phase HPLC using a XSelect HSS T3 5 μm 4.6 mm × 150 mm Column (Waters) in the HP Agilent 1100 series (Hewlett-Packard) liquid chromatograph. Then we used an XSelect Column HSS T3 OBD Prep Colum 100 Å 5 μm 10 mm × 150 mm with a flow rate of 4.7 mL min^–1^ in 0.1% ammonium formate for the purification rounds. Injection volume per run was ∼50 μL, and the fractions corresponding to peaks detected by UV–vis diode at 300–350 nm were collected and pooled after each run. The Turb Vap LV Evaporator at 28 °C was used to evaporate the solvents in the fractions, and the water was removed by lyophilization as described previously. The purified MAA samples were then confirmed by HR-LCMS and sent for NMR analysis.

### NMR Analysis of Purified MAA Chemical Variants

The two purified MAA chemical variants were then dried and sent for NMR analysis. All NMR spectra for the samples were collected using a Bruker Avance III HD 800 MHz NMR spectrometer, equipped with a cryogenically cooled, z-gradient TCI ^1^H, ^13^C, ^15^N triple resonance probehead. Data were collected at 298 K in D_2_O. 582 Da MAA (aplysiapaltyhine E) hexoses were identified as described previously.^42^

### Whole Genome Sequencing of the *Nostoc* sp. UHCC 0926

A four-week-old 40 mL nonaxenic culture of *Nostoc* sp. UHCC 0926 was harvested by centrifuging at 7000 g for 5 min and the pellets were washed three times with 45 mL of sterile Z8 media to reduce contamination, as the culture was not axenic. The DNA extraction was performed with a standard phenol-chloroform and ethanol precipitation method. Extracted DNA was dissolved in 30 μL of 5 mM TrisHCl at pH 8. DNA quantity and quality were assessed using Nanodrop 1 spectrophotometer (Thermo Fisher Scientific).

Pacbio Sequel II instrument was used for the sequencing reactions, and the initial assemblies were done according to the Pacbio’s SMRTlink version 9 microbial assembly user guide at the University of Helsinki sequencing center. The 573,523 subreads containing 8,537,413,497 bp were assembled into 490 contigs and 33.8 Mbps of sequence. Sequence circularity was checked with GAP4 (Staden package), and minimap2 was used for mapping Pacbio HiFi reads (37,287 reads, containing 628,850,915 bp) back to assembly and then polishing genome using Pilon (v 1.16). *De novo* genome assemblies were obtained with Flye 2.9. The assembled scaffolds were classified with Kaiju 1.7.2 at the phylum level and separated using in-house scripts to obtain only cyanobacterial scaffolds. The circularity of sequences was checked with Bandage 0.8.1 and the completeness and contamination of the genomes were assessed with CheckM 1.0.13.^43^ The complete genome can be accessed from the NCBI GenBank database with accession number PRJNA930330.

### Identification of MAA Biosynthetic Gene Clusters

We collected all of the obtained genome sequence FASTA files in a custom database in a UNIX environment. Amino acid sequences of the MAA biosynthetic enzymes from *Trichormus variabilis* ATCC 29413 and *Nostoc punctiforme* ATCC 29133 were used as references for tBLASTn 2.2.31+ alignments against the sequences in this custom genome database.^44^ Here we identified the strains that had MAA biosynthetic gene clusters based on the percentage identity hits of ≥50% to the reference sequences.

### Phylogenetic Distribution of the MAA Biosynthetic Enzymes in Cyanobacterial Genomes

To investigate whether discontiguous MAA biosynthetic gene clusters are common in cyanobacterial genomes, we used MAA biosynthetic enzyme sequences from Nostoc sp. UHCC 0926 as reference sequences to identify similar organizations in 293 complete cyanobacterial genomes (including plasmids) deposited in the NCBI accessed on February 18, 2022. Protein alignments were performed with BLASTp 2.8.1+^45^ considering minimum identity values of 45% and minimum coverage values of 75% for the established MysABCDE amino acid sequences.^14^ The additional putative MAA biosynthetic genes were only considered part of the cluster when they were located below the 10 kb flanking region of established MAA biosynthetic genes. The modular architecture and substrate specificity of the MysE enzymes were analyzed using antiSMASH/NRPSPredictor2.^46^

The maximum-likelihood phylogenomic tree of the complete genomes with MAA biosynthetic gene clusters identified was inferred with RAxML v 8.0.0^47^ with 1000 bootstraps using the PROTGAMMAIGTR model. 120 bacterial single-copy conserved marker proteins’ sequences were aligned with GTDB-Tk v 0.3.2.^48^ The maximum-likelihood phylogenetic tree based on MAA biosynthetic proteins were done by aligning the amino acid sequences with MUSCLE^49^ and inferring the trees with FastTree 2.1.11^50^ with WAG+GAMMA models. All the trees were visualized and edited with iTOL.^51^

## Supporting Information Available

HR-LCMS MS^E^ chromatograms of all the detected MAA structural variants along with full and partially annotated ^1^H NMR, TOCSY, COSY, HSQC, and HMBC spectra for the tricore B and aplysiapalythine E. Also included are the whole genome assembly stats of *Nostoc* sp. UHCC 0926, table of MAA biosynthetic enzymes identified, and the phylogenetic tree figures constructed for the rest of the MAA biosynthetic gene cluster enzymes (PDF)

## References

[ref1] Garcia-Pichel F.; Castenholz R. W. Occurrence of UV-absorbing, mycosporine-like compounds among cyanobacterial isolates and an estimate of their screening capacity. Appl. Environ. Microbiol. 1993, 59, 163–169. 10.1128/aem.59.1.163-169.1993. 16348839 PMC202072

[ref2] Carreto J. I.; Carignan M. O. Mycosporine-like amino acids: relevant secondary metabolites. Chemical and ecological aspects. Mar Drugs 2011, 9, 387–446. 10.3390/md9030387. 21556168 PMC3083659

[ref3] Jain S.; et al. Cyanobacteria as efficient producers of mycosporine-like amino acids. J. Basic Microbiol 2017, 57, 715–727. 10.1002/jobm.201700044. 28543536

[ref4] Martins T. P.; Arsin S.; Fewer D. P.; Leão P. UV-protective secondary metabolites from cyanobacteria. Pharmacological Potential of Cyanobacteria 2022, 107–144. 10.1016/B978-0-12-821491-6.00005-3.

[ref5] Koizumi K.; et al. How seaweeds release the excess energy from sunlight to surrounding sea water. Phys. Chem. Chem. Phys. 2017, 19, 15745–15753. 10.1039/C7CP02699D. 28604867

[ref6] Sakamoto T.; et al. The extracellular-matrix-retaining cyanobacterium *Nostoc verrucosum* accumulates trehalose but is sensitive to desiccation. FEMS Microbiol Ecol 2011, 77, 385–394. 10.1111/j.1574-6941.2011.01114.x. 21507024

[ref7] Wada N.; Sakamoto T.; Matsugo S. Mycosporine-Like Amino Acids and Their Derivatives as Natural Antioxidants. Antioxidants (Basel) 2015, 4, 603–46. 10.3390/antiox4030603. 26783847 PMC4665425

[ref8] Arbeloa E. M.; Bertolotti S. G.; Churio M. S. Photophysics and reductive quenching reactivity of gadusol in solution. Photochemical and Photobiological Sciences 2011, 10, 133–142. 10.1039/c0pp00250j. 21072419

[ref9] Moliné M.; et al. UVB Photoprotective Role of Mycosporines in Yeast: Photostability and Antioxidant Activity of Mycosporine-Glutaminol-Glucoside. Radiat. Res. 2011, 175, 44–50. 10.1667/RR2245.1. 21175346

[ref10] Matsuyama K.; et al. PH-Independent Charge Resonance Mechanism for UV Protective Functions of Shinorine and Related Mycosporine-like Amino Acids. J. Phys. Chem. A 2015, 119, 12722–12729. 10.1021/acs.jpca.5b09988. 26625701

[ref11] Chrapusta E.; Kaminski A.; Duchnik K.; Bober B.; Adamski M.; Bialczyk J. Mycosporine-Like Amino Acids: Potential Health and Beauty Ingredients. Mar Drugs 2017, 15 (10), 326 10.3390/md15100326. 29065484 PMC5666432

[ref12] Llewellyn C. A.; Airs R. L. Distribution and abundance of MAAs in 33 species of microalgae across 13 classes. Mar Drugs 2010, 8, 1273–91. 10.3390/md8041273. 20479978 PMC2866486

[ref13] Geraldes V.; Pinto E. Mycosporine-Like Amino Acids (MAAs): Biology, Chemistry and Identification Features. Pharmaceuticals 2021, 14, 63 10.3390/ph14010063. 33466685 PMC7828830

[ref14] Balskus E. P.; Walsh C. T. The genetic and molecular basis for sunscreen biosynthesis in cyanobacteria. Science 2010, 329, 1653–6. 10.1126/science.1193637. 20813918 PMC3116657

[ref15] Fischbach M. A.; Walsh C. T.; Clardy J. The evolution of gene collectives: How natural selection drives chemical innovation. Proc. Natl. Acad. Sci. U. S. A. 2008, 105, 4601–4608. 10.1073/pnas.0709132105. 18216259 PMC2290807

[ref16] Shang J. L.; et al. UV-B induced biosynthesis of a novel sunscreen compound in solar radiation and desiccation tolerant cyanobacteria. Environ. Microbiol 2018, 20, 200–213. 10.1111/1462-2920.13972. 29076601

[ref17] Chen M.; Rubin G. M.; Jiang G.; Raad Z.; Ding Y. Biosynthesis and Heterologous Production of Mycosporine-Like Amino Acid Palythines. J. Org. Chem. 2021, 86, 11160–11168. 10.1021/acs.joc.1c00368. 34006097 PMC8905528

[ref18] Nazifi E.; et al. Characterization of the chemical diversity of glycosylated mycosporine-like amino acids in the terrestrial cyanobacterium *Nostoc commune*. J. Photochem. Photobiol. B 2015, 142, 154–168. 10.1016/j.jphotobiol.2014.12.008. 25543549

[ref19] Ishihara K.; et al. Novel glycosylated mycosporine-like amino acid, 13-O-(β-galactosyl)-porphyra-334, from the edible cyanobacterium *Nostoc sphaericum* -protective activity on human keratinocytes from UV light. J. Photochem. Photobiol. B 2017, 172, 102–108. 10.1016/j.jphotobiol.2017.05.019. 28544967

[ref20] Agrawal P. K. NMR Spectroscopy in the structural elucidation of oligosaccharides and glycosides. Phytochemistry 1992, 31, 3307–3330. 10.1016/0031-9422(92)83678-R. 1368855

[ref21] Geraldes V.; Jacinavicius F. R.; Genuário D. B.; Pinto E. Identification and distribution of mycosporine-like amino acids in Brazilian cyanobacteria using ultrahigh-performance liquid chromatography with diode array detection coupled to quadrupole time-of-flight mass spectrometry. Rapid Commun. Mass Spectrom. 2020, 34, S3 10.1002/rcm.8634. 31677357

[ref22] Kicklighter C. E.; Kamio M.; Nguyen L.; Germann M. W.; Derby C. D. Mycosporine-like amino acids are multifunctional molecules in sea hares and their marine community. Proc. Natl. Acad. Sci. U. S. A. 2011, 108, 11494–11499. 10.1073/pnas.1103906108. 21709250 PMC3136258

[ref23] Kamio M.; Kicklighter C. E.; Nguyen L.; Germann M. W.; Derby C. D. Isolation and Structural Elucidation of Novel Mycosporine-Like Amino Acids as Alarm Cues in the Defensive Ink Secretion of the Sea Hare *Aplysia californica*. Helv. Chim. Acta 2011, 94, 1012–1018. 10.1002/hlca.201100117.

[ref24] Matsui K.; et al. Novel glycosylated mycosporine-like amino acids with radical scavenging activity from the cyanobacterium *Nostoc commune*. J. Photochem. Photobiol. B 2011, 105, 81–89. 10.1016/j.jphotobiol.2011.07.003. 21813286

[ref25] Spence E.; Dunlap W. C.; Shick J. M.; Long P. F. Redundant Pathways of Sunscreen Biosynthesis in a Cyanobacterium. ChemBioChem. 2012, 13, 531–533. 10.1002/cbic.201100737. 22278966

[ref26] Pope M. A.; et al. O-Methyltransferase Is Shared between the Pentose Phosphate and Shikimate Pathways and Is Essential for Mycosporine-Like Amino Acid Biosynthesis in *Anabaena variabilis* ATCC 29413. ChemBioChem. 2015, 16, 320–327. 10.1002/cbic.201402516. 25487723

[ref27] Geraldes V.; et al. Genetic and biochemical evidence for redundant pathways leading to mycosporine-like amino acid biosynthesis in the cyanobacterium *Sphaerospermopsis torques-reginae* ITEP-024. Algae 2020, 35, 177–187. 10.4490/algae.2020.35.5.19.

[ref28] Mogany T.; Kumari S.; Swalaha F. M.; Bux F. In silico analysis of enzymes involved in mycosporine-like amino acids biosynthesis in *Euhalothece* sp.: Structural and functional characterization. Algal Res. 2022, 66, 102806 10.1016/j.algal.2022.102806.

[ref29] Zhang Z. C.; et al. New types of ATP-grasp ligase are associated with the novel pathway for complicated mycosporine-like amino acid production in desiccation-tolerant cyanobacteria. Environ. Microbiol 2021, 23, 6420–6432. 10.1111/1462-2920.15732. 34459073

[ref30] Nazifi E.; et al. Glycosylated Porphyra-334 and Palythine-Threonine from the Terrestrial Cyanobacterium *Nostoc commune*. Mar Drugs 2013, 11, 3124–3154. 10.3390/md11093124. 24065157 PMC3801118

[ref31] D’Agostino P. M.; et al. Comparative Profiling and Discovery of Novel Glycosylated Mycosporine-Like Amino Acids in Two Strains of the Cyanobacterium *Scytonema cf. crispum*. Appl. Environ. Microbiol. 2016, 82, 5951–9. 10.1128/AEM.01633-16. 27474710 PMC5038028

[ref32] Wright D. J.; et al. UV irradiation and desiccation modulate the three-dimensional extracellular matrix of *Nostoc commune* (Cyanobacteria). J. Biol. Chem. 2005, 280, 40271–81. 10.1074/jbc.M505961200. 16192267

[ref33] Komárek J. A polyphasic approach for the taxonomy of cyanobacteria: principles and applications. European Journal of Phycology 2016, 51, 346–353. 10.1080/09670262.2016.1163738.

[ref34] Osborn A. R.; et al. De novo synthesis of a sunscreen compound in vertebrates. Elife 2015, 4, 5 10.7554/eLife.05919. PMC4426668 25965179

[ref35] Gao Q.; Garcia-Pichel F. An ATP-grasp ligase involved in the last biosynthetic step of the iminomycosporine shinorine in *Nostoc punctiforme* ATCC 29133. J. Bacteriol. 2011, 193, 5923–8. 10.1128/JB.05730-11. 21890703 PMC3194895

[ref36] Bosello M.; Robbel L.; Linne U.; Xie X.; Marahiel M. A. Biosynthesis of the siderophore rhodochelin requires the coordinated expression of three independent gene clusters in *Rhodococcus jostii* RHA1. J. Am. Chem. Soc. 2011, 133, 4587–4595. 10.1021/ja1109453. 21381663

[ref37] Fewer D. P.; Metsä-Ketelä M. A pharmaceutical model for the molecular evolution of microbial natural products. FEBS Journal 2020, 287, 1429–1449. 10.1111/febs.15129. 31693795

[ref38] Demoulin C. F.; et al. Cyanobacteria evolution: Insight from the fossil record. Free Radic Biol. Med. 2019, 140, 206–223. 10.1016/j.freeradbiomed.2019.05.007. 31078731 PMC6880289

[ref39] Vanhaelewyn L.; van der Straeten D.; de Coninck B.; Vandenbussche F. Ultraviolet Radiation from a Plant Perspective: The Plant-Microorganism Context. Front Plant Sci. 2020, 11, 12 10.3389/fpls.2020.597642. 33384704 PMC7769811

[ref40] Chen M. Y.; et al. Comparative genomics reveals insights into cyanobacterial evolution and habitat adaptation. ISME Journal 2021, 15, 211–227. 10.1038/s41396-020-00775-z. 32943748 PMC7852516

[ref41] Kotai J. Instructions for preparation of modified nutrient solution Z8 for algae. Norwegian Institute for Water Research, Oslo 1972, 11, 5 10.1127/0029-5035/2004/0079-0099.

[ref42] Heinilä L. M. P.; et al. Discovery of varlaxins, new aeruginosin-type inhibitors of human trypsins. Org. Biomol Chem. 2022, 20, 2681–2692. 10.1039/D1OB02454J. 35293909

[ref43] Parks D. H.; Imelfort M.; Skennerton C. T.; Hugenholtz P.; Tyson G. W. CheckM: assessing the quality of microbial genomes recovered from isolates, single cells, and metagenomes. Genome Res. 2015, 25, 1043–1055. 10.1101/gr.186072.114. 25977477 PMC4484387

[ref44] Altschul S. F.; Gish W.; Miller W.; Myers E. W.; Lipman D. J. Basic local alignment search tool. J. Mol. Biol. 1990, 215, 403–410. 10.1016/S0022-2836(05)80360-2. 2231712

[ref45] Camacho C.; et al. BLAST+: Architecture and applications. BMC Bioinformatics 2009, 10, 1–9. 10.1186/1471-2105-10-421. 20003500 PMC2803857

[ref46] Blin K.; et al. AntiSMASH 6.0: Improving cluster detection and comparison capabilities. Nucleic Acids Res. 2021, 49, W29–W35. 10.1093/nar/gkab335. 33978755 PMC8262755

[ref47] Stamatakis A. RAxML version 8: a tool for phylogenetic analysis and post-analysis of large phylogenies. Bioinformatics 2014, 30, 1312–1313. 10.1093/bioinformatics/btu033. 24451623 PMC3998144

[ref48] Chaumeil P. A.; Mussig A. J.; Hugenholtz P.; Parks D. H. GTDB-Tk: a toolkit to classify genomes with the Genome Taxonomy Database. Bioinformatics 2020, 36, 1925–1927. 10.1093/bioinformatics/btz848. PMC7703759 31730192

[ref49] Edgar R. C. MUSCLE: multiple sequence alignment with high accuracy and high throughput. Nucleic Acids Res. 2004, 32, 1792–1797. 10.1093/nar/gkh340. 15034147 PMC390337

[ref50] Price M. N.; Dehal P. S.; Arkin A. P. FastTree 2 - Approximately Maximum-Likelihood Trees for Large Alignments. PLoS One 2010, 5, e9490 10.1371/journal.pone.0009490. 20224823 PMC2835736

[ref51] Letunic I.; Bork P. Interactive Tree of Life (iTOL) v4: recent updates and new developments. Nucleic Acids Res. 2019, 47, W256–W259. 10.1093/nar/gkz239. 30931475 PMC6602468

