# Supplementary material for: A Plastic Biosynthetic Pathway for the Production of Structurally Distinct Microbial Sunscreens
Source: ACS Chem Biol. 2023 Aug 21;18(9):1959–67. doi: 10.1021/acschembio.3c00112 (PMC10510106; doi:10.1021/acschembio.3c00112)
Supplement: Supplementary file 1 — cb3c00112_si_001.pdf [file cb3c00112_si_001.pdf]

Supporting Information

A plastic biosynthetic pathway for the production of structurally distinct microbial sunscreens

Sıla Arsın[1], Endrews Delbaje[2], Jouni Jokela[1], Matti Wahlsten[1], Zoë M. Farrar[1], Perttu Permi[3, 4], David Fewer[1*]

[1] University of Helsinki, Department of Microbiology, Faculty of Agriculture and Forestry, 00014 Helsinki, Finland.
[2] University of São Paulo, Center for Nuclear Energy in Agriculture, Avenida Centenário 303, 13400-970 Piracicaba, SP, Brazil
[3]Department of Chemistry, University of Jyväskylä, 40014 Jyväskylä, Finland
[4]Department of Biological and Environmental Science, Nanoscience Center, University of Jyväskylä, 40014 Jyväskylä, Finland

*Corresponding author
E-mail address: david.fewer@helsinki.fi

Table of Contents

Table S1. MAAs variants detected from the *Nostoc* sp. UHCC 0926 extract S2
Figure S1. Ion chromatograms of the 22 MAA variant and intermediate structures..S3
Figure S2. MS^E collision spectra of the ion chromatogram peaks for the MAA structures 1 to 11 ....S4
Figure S3. MS^E collision spectra of the ion chromatogram peaks for the MAA structures 12 to 22 ....S5
NMR Parameters S6
Table S2. NMR data of aplysiapalythine E (**19**) sample in D_2O S7
Table S3 Aplysiapalythine E (**19**) product ion data from MS^E spectrum data S8
Table S4 NMR data for Tricore (**18**) B in D_2O S9
Figure S4. Partly annotated proton spectrum of aplysiapalythine E (**19**) sample in D_2O S10
Figure S5. Annotated carbon spectrum of aplysiapalythine E (**19**) sample in D_2O S10
Figure S6. Partly annotated ^1H-^1H TOCSY (90 ms) spectrum of aplysiapalythine E (**19**) S11
Figure S7. Partly annotated ^1H-^1H COSY spectrum of aplysiapalythine E (**19**) S12
Figure S8 Partly annotated ^1H-^{13}C HSQC-TOCSY spectrum of aplysiapalythine E (**19**) S13
Figure S9. Partly annotated edited ^1H-^{13}C HSQC spectrum of aplysiapalythine E (**19**) S14
Figure S10. ^1H-^{13}C HMBC spectrum of aplysiapalythine E (**19**) S15
Figure S11. Partial ^1H spectra of aplysiapalythine E (**19**) acid hydrolysate in 2 M D_2SO_4 (in D_2O) S16
Figure S12. Product ions of aplysiapalythine E (**19**) from MS^E spectrum S16
Figure S13. Annotated proton spectrum of tricore B (**18**) in D_2O..S17
Figure S14. Annotated carbon spectrum of tricore B (**18**) in D_2O S17
Figure S15. Annotated ^1H-^1H TOCSY (90 ms) spectrum of tricore B (**18**) in D_2O S18
Figure S16. Annotated ^1H-^1H COSY spectrum of tricore B (**18**) in D_2O..S19
Figure S17. Annotated 1H-13C HSQC spectrum of tricore B (**18**) in D2O S19

Figure S18. Annotated ^{1}H-^{13}C HMBC spectrum of tricore B (**18**) in D_2O S20
Table S5. DNA size break down of *Nostoc* sp. UHCC 0926 complete genome assembly S20
Table S6. MAA biosynthetic gene cluster enzymes of *Nostoc* sp. UHCC 0926 ....S21
Figure S19. Phylogenetic tree constructed using MysA protein sequences S22
Figure S20. Phylogenetic tree constructed using MysB & MysF protein sequences S23
Figure S21. Phylogenetic tree constructed using MysC protein sequences S24
Figure S22. Phylogenetic tree constructed using MysD protein sequences S25
Figure S23. Phylogenetic tree constructed using MysH & MysI protein sequences S26

Table S1. Substructures, retention times (t_R) and calculated *m/z* values of protonated MAA variants 1 – 22 from *Nostoc* sp. UHCC 0926. Difference of measured *m/z* value to the calculated *m/z* value (Δ, red if > ± 5) in parts per million (ppm) and peak areas of variants (relative areas in %). Structures of aminocyclohexenone (ACH) and aminocyclohexenimine (ACHI) at the top of the table. The values corresponding to tricore B **(18)** and aplysiapalythine E **(19)** are bolded which make up the 17.74 % and 52.08 % of the total MAA intermediates respectively. Intermediate structures included in the biosynthetic scheme (Figure 2) are highlighted with a grey background.

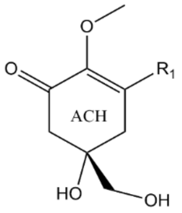

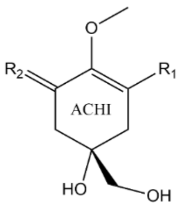

	Structure						t_R	*m/z*	Δ	Area	
No	ACH	ACHI	R_1	R_2	R_2	Hex	(min)	Calc	(ppm)	units	(%)
1	-	1	Gly	$NHCH_3$	-	-	5.80	259.12885	-0.4	31709	12.50
2	-	1	Gly	NH_2	-	-	5.91	245.11320	-2.7	201	0.08
3	1	-	Gly	-	-	-	5.91	246.09721	3.4	184	0.07
4	-	1	Ser	NH_2	-	-	6.44	275.12376	-2.2	48	0.02
5	2	-	Orn	-	-	-	7.04	473.21297	-0.7	142	0.06
6	1	1	Orn	NH_2	-	-	7.15	472.22896	0.4	1011	0.40
7	1	1	Orn	NH_2	-	-	7.25	472.22896	1.0	1134	0.45
8	-	1	Gly	Ala	-	-	7.37	317.13433	-0.6	2042	0.80
9	-	1	Gly	Thr	-	-	7.56	347.14489	-3.3	43	0.02
10	-	1	Gly	$NHCH_3$	-	1	7.56	421.18167	0.2	30904	12.18
11	1	-	Orn	-	-	-	7.66	303.15506	-2.0	587	0.23
12	-	1	Gly	Ser	-	-	7.67	333.12924	-3.9	23	0.01
13	-	1	Gly	NH_2	-	1	7.73	407.16602	-1.9	80	0.03
14	-	2	Orn	Gly	Orn	-	8.01	644.31375	-10.1	51	0.02
15	-	1	Orn	NH_2	-	-	8.13	302.17105	7.6	2083	0.82
16	1	1	Orn	Gly	-	-	8.22	530.23444	0.0	872	0.34
17	-	2	Orn	Gly	Gly	-	8.55	587.25590	8.6	80	0.03
**18**	**2**	**1**	**Orn**	**Orn**	**-**	**-**	**8.70**	**757.35019**	**3.4**	**45011**	**17.74**
**19**	**-**	**1**	**Gly**	**$NHCH_3$**	**-**	**2**	**8.94**	**583.23450**	**1.3**	**132141**	**52.08**
20	1	1	Orn	Orn	-	-	9.24	587.29228	-0.1	5205	2.05
21	2	1	Orn	Orn	-	1	9.37	919.40302	-0.6	69	0.03
22	-	1	Orn	Orn	-	-	10.08	417.23438	-2.5	94	0.04

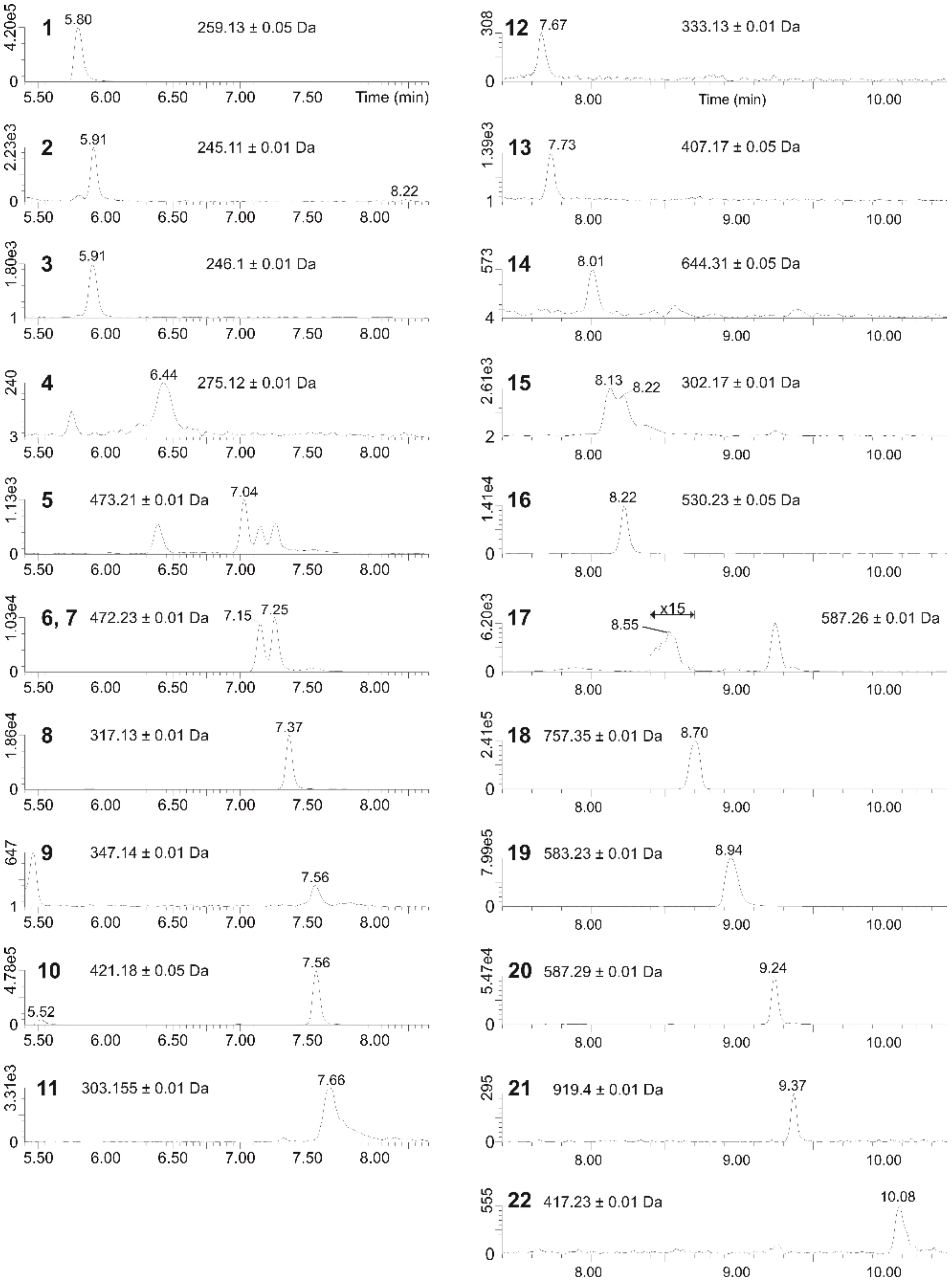

Figure S1. Extracted ion chromatograms of MAA variants 1 – 22.

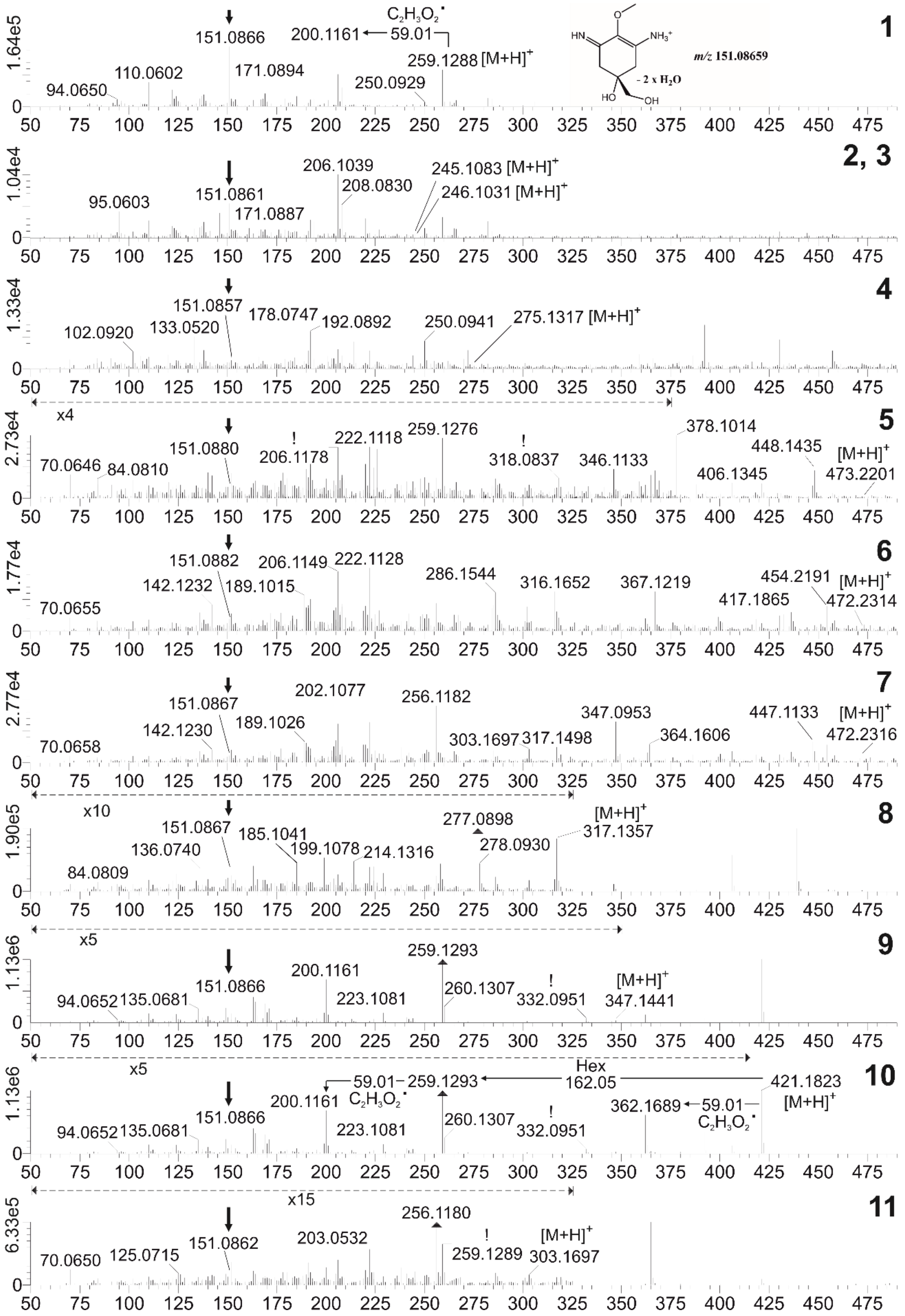

Figure S2. MS^E (E: elevated collision energy) spectra of the extracted ion chromatogram peaks representing MAA variants 1 – 11 presented in Figure S1. Dotted line with arrow ends shows the magnified range of the spectrum and the x-number the magnification. Fragment *m/z* 151.08659 pointed with an arrow is diagnostic for MAAs.

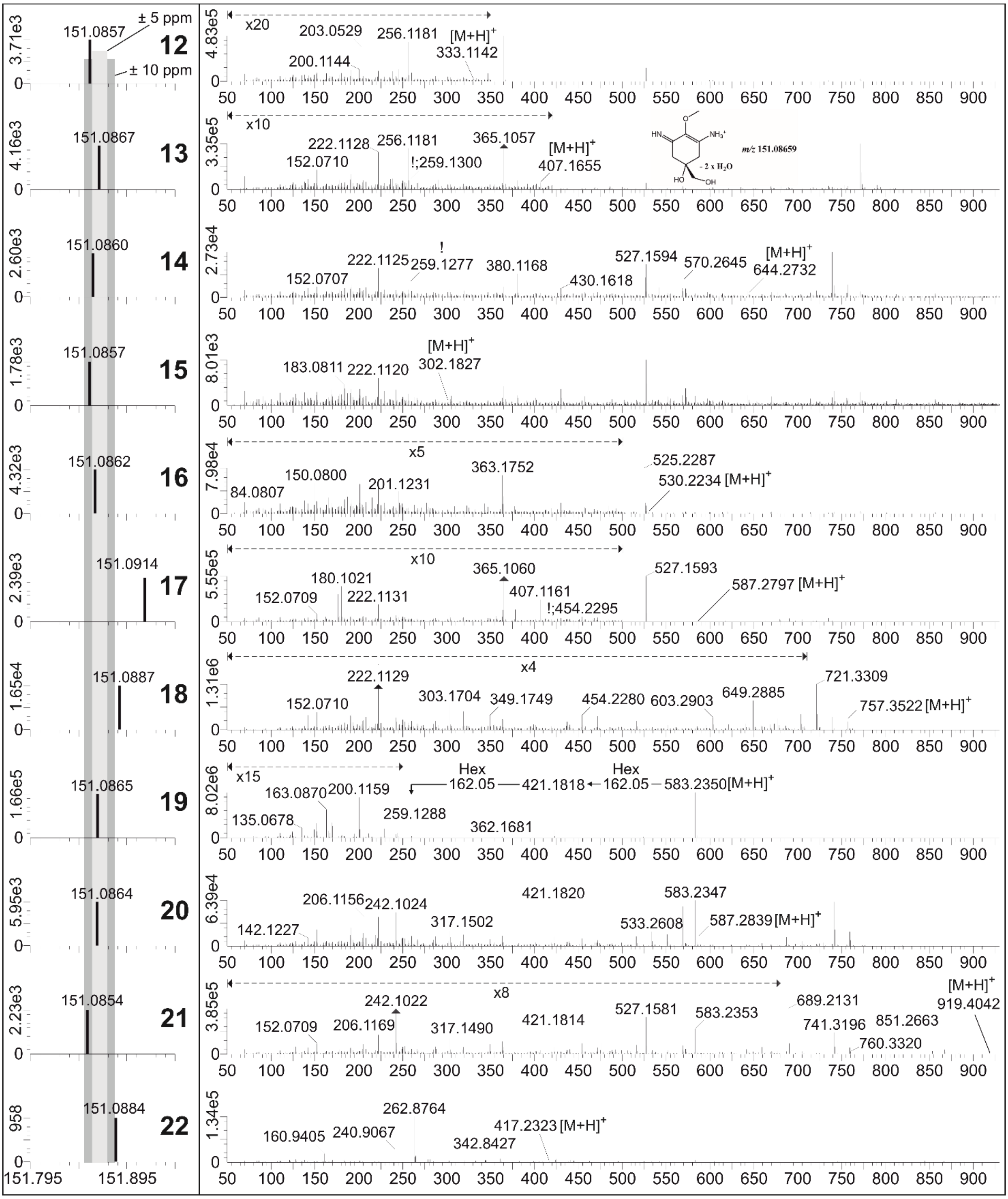

Figure S3. MS^E (E: elevated collision energy) spectra of the extracted ion chromatogram peaks representing MAAvariants 12 – 22 presented in Figure S1. Dotted line with arrow ends shows the magnified range of the spectrum and the x-number the magnification. Fragment *m/z* 151.08659 shown in left panel is diagnostic for MAAs.

NMR Parameters

To obtain assignment of ^{1}H and ^{13}C resonances in MAAs, a set of two-dimensional homonuclear and heteronuclear NMR experiments were collected in addition to the conventional ^{1}H presaturation and ^{13}C-{^{1}H} experiments. Total correlation spectroscopy (TOCSY, mixing time of 90 ms) and double-quantum filtered correlation spectroscopy (DQF-COSY) experiments were employed to identify spin-systems. Heteronuclear single quantum coherence (^{13}C HSQC and spin-multiplicity edited ^{13}C HSQC) and heteronuclear multiple bond correlation (^{13}C HMBC) experiments were used to assign ^{1}H and ^{13}C one-bond and multiple-bond connectivities. ^{1}H experiment, with residual water presaturation during T_1 recovery delay (2 s), was collected with 32k complex points, corresponding to acquisition time of 1.28 s. The signal was accumulated with 4 scans. ^{13}C-{^{1}H} spectrum was collected using 96k complex points, corresponding acquisition time of 1s. Recycle delay was 3 seconds during which gated ^{1}H decoupling was applied. Two-dimensional TOCSY spectrum was collected with 200 and 4100 complex points in t_1 and t_2, resulting in 15.6 ms and 320 ms acquisition times. TOCSY spectrum was measured with the isotropic mixing time of 90 ms using DIPSI-2 spin-lock. Number of transients was 8 and T_1 recovery time set to 1 s. Two-dimensional DQF-COSY spectrum was collected using 300 and 2048 complex points in t_1 and t_2, corresponding to acquisition times of 18.7 ms and 128 ms, respectively. ^{13}C HSQC experiments with (and without) spin-multiplicity editing and utilizing gradient-enhanced sensitivity improvement scheme, were collected using 2 transients with 512 (400) and 2048 complex points in t_1 and t_2, respectively. These correspond to acquisition times of 7 (5.5) ms and 160 (92) ms, respectively. The T_1 recovery delay of 1.5 was used. The ^{13}C HMBC spectrum was collected using 1024 and 4096 points in t_1 and t_2, respectively. This translates to acquisition times of 11 ms and 180 ms, respectively. Signal was accumulated with 12 transients using recycle delay of 1.18 seconds. The transfer delay for long-range correlations was set to 8 Hz**.**

Table S2. NMR data of aplysiapalythine E (**19**) (582 Da) sample in D_2O. Myc A and B are the two detected MAA core units and Hex1 and 2 are the two main hexose units detected. **δ_C** values are from 1D ^{13}C and ^{1}H-^{13}C HMBC spectra.

No	δ_H	mult., *J*(Hz)	δ_C	COSY	TOCSY	HSQC-TOCSY	HMBC
**Myc A**							
1	-		163.5				
2	-		125.9				
3	-		162.9				
4a*	2.73	17.21	36.2	4b	4b	4	1, 2, 3, 5, 6, 7
4b*	3.00	-		4a	4a	4	(1), 3, (5)
5	-		69.8				
6*	2.97	18.10	37.9			6	
7a	3.68		75.2			7	Hex1/2-1
7b	3.92					7	
8	3.50		60.4			8	2
**Myc B**							
1	-		163.8				
2	-		125.9				
3	-		163.1				
4a**	2.66	16.82	35.9	4b	4b	4	1, 2, 3, 5, 6, 7
4b**	2.90	16.8		4a	4a	4	(3), (5)
5	-		70.5				
6**	2.89		37.7			6	
7	3.56		67.4			7	
8	3.50		60.4			8	2
**Hex1**							
1	4.42	7.83	103.3	2	2, 3, 4	1, 2, 3, 4	A-7, 3, (A-5), 6
2	3.50		70.7	1, 3	1, 3	1, 2, 3, 4	1, 3
3	3.62		72.7	2, 4	1, 2, 4	1, 2, 3, 4	2, 4
4	3.90		68.6	3	1, 2, 3	1, 2, 3, 4	1, 2, 3, 5
5	3.88		73.8			5, 6	1, 6
6a	3.88		69.1			5, 6	5
6b	4.04					5, 6	1, (4)
**Hex2**							
1	4.43	7.83	103.4	2	2, 3, 4		(B-5)
2	3.56		70.7	1, 3	1, 3	1, 2, 4	-
3	3.65		72.5	2, 4	1, 2, 4	1, (2), 3, 4	2
4	3.96		68.6	3	1, 2, 3	1, 2, 3, 4	2, 3, (5)
5							
6a							
6b							

*, ** = 4/4' and 6 signals can be interchanged.

Table S3. Product ion data of from MS^E spectrum of aplysiapalythine E variant's (**19**) chromatographic peak. Codes are marked to the aplysiapalythine E in Figure S3 Δ = difference of calculated (Calc) and experimental (Exp) ion masses in parts per million (ppm).

**Product ion**			***m/z***		
**code**		**Neutral loss**	**Calc**	**Exp**	**□ (ppm)**
	$[M+H]^{+}$		583.23450	583.23575	2.1
a	$[M+H]^{+}$	$C_2H_2O_2$	525.22902	525.22835	-1.3
a	$[M+H]^{+}$	$C_2H_3O_2^{\bullet}$	524.22119	524.22205	1.6
b	$[M+H]^{+}$	$C_4H_8O_4$	463.19224	463.19295	1.5
c	$[M+H]^{+}$	Glc	421.18167	421.18225	1.4
c-d	$[M+H]^{+}$	Glc, CH_2O	391.17111	391.17035	-1.9
a-c	$[M+H]^{\bullet+}$	Glc, $C_2H_3O_2^{\bullet}$	362.16837	362.16795	-1.2
a-c	$[M+H]^{+}$	Glc, $C_2H_4O_2$	361.16054	361.15995	-1.6
e	$[M+H]^{+}$	Glc-Gal	259.12885	259.12885	0.0
e-f	$[M+H]^{\bullet+}$	Glc-Gal, $CH_3^{\bullet}$	244.10537	244.10525	-0.5
e	$[M+H]^{+}$	Glc-Gal, H_2O	241.11828	241.11835	0.3
e-d	$[M+H]^{+}$	Glc-Gal, CH_2O	229.11828	229.11805	-1.0
e	$[M+H]^{+}$	Glc-Gal, $2xH_2O$	223.10772	223.10765	-0.3
a-e	$[M+H]^{\bullet+}$	Glc-Gal, $C_2H_3O_2^{\bullet}$	200.11554	200.11585	1.5
a-e	$[M+H]^{+}$	Glc-Gal, CH_3COOH	199.10772	199.10785	0.7
a-d-e	$[M+H]^{+}$	Glc-Gal, CH_3COOH, CH_2O	169.09715	169.09735	1.2
a-e	$[M+H]^{\bullet+}$	Glc-Gal, $C_2H_3O_2^{\bullet}$, $2xH_2O$	164.09441	164.09405	-2.2
a-e	$[M+H]^{+}$	Glc-Gal, CH_3COOH, $2xH_2O$	163.08659	163.08695	2.2
a-d-e	$[M+H]^{+}$	Glc-Gal, CH_3COOH, CH_2O, H_2O	151.08659	151.08685	1.7

Table S4. NMR data of the 756 Da tricore B (**18**) sample in D_2O.

No	δ_H	δ_C	HMBC
1		161.9-162.2	
2		128.59	
3		161.9-162.2	
4	2.75[a]	36.1	2, 3, 5, 6, 7, 8
4'	2.90[a]		2, 3, 5, 6, 7
5		74.01	
6	2.78[a]	36.1	1, 2, 4, 5, 7
6'	2.80[a]		1, 2, 4, 5, 7
7	3.56	70.43	2, 4, 5
7			
8	3.65	62.29	2
9	3.39	45.15	3, 10, 11
10	1.65	29.08	9, 11, 12
11	1.92	32.41	9, 10, 12, 13
12	4.20	61.50	10, 11, 13, 14
13		179.76	
14		161.9-162.2	
15		132.9	
16		187.5	
17	2.37	45.8	15, 16, 18, 19, 20
17'	2.63		15, 16, 18, 19, 20, 21
18		75.1	
19	2.75	35.69	14, 15, 17, 18, 20
19'	2.84		
20	3.53	70.6	17, 18
21	3.55	62.12	15
22	3.41	45.18	1, 23, 24
23	1.71	29.31	22, 24, 25
24	1.99	32.46	22, 23, 25, 26
25	4.20	61.58	23, 24, 26, 27
26		179.76	
27		161.9-162.2	
28		132.9	
29		187.5	
30	2.38	45.8	28, 29, 31, 32, 33
30'	2.66		28, 29, 31, 32, 33, 34
31		75.1	
32	2.78	35.69	27, 28, 30, 31, 33
32'	2.85		27, 28, 30, 31, 33
33	3.53	70.6	30, 31
34	3.55	62.12	28

J(Hz) = 17.2 for protons 4, 4’, 6’, 17, 17’, 19’, 30, 30’, 32’ [a] = 4/4' and 6/6' could be other way around.

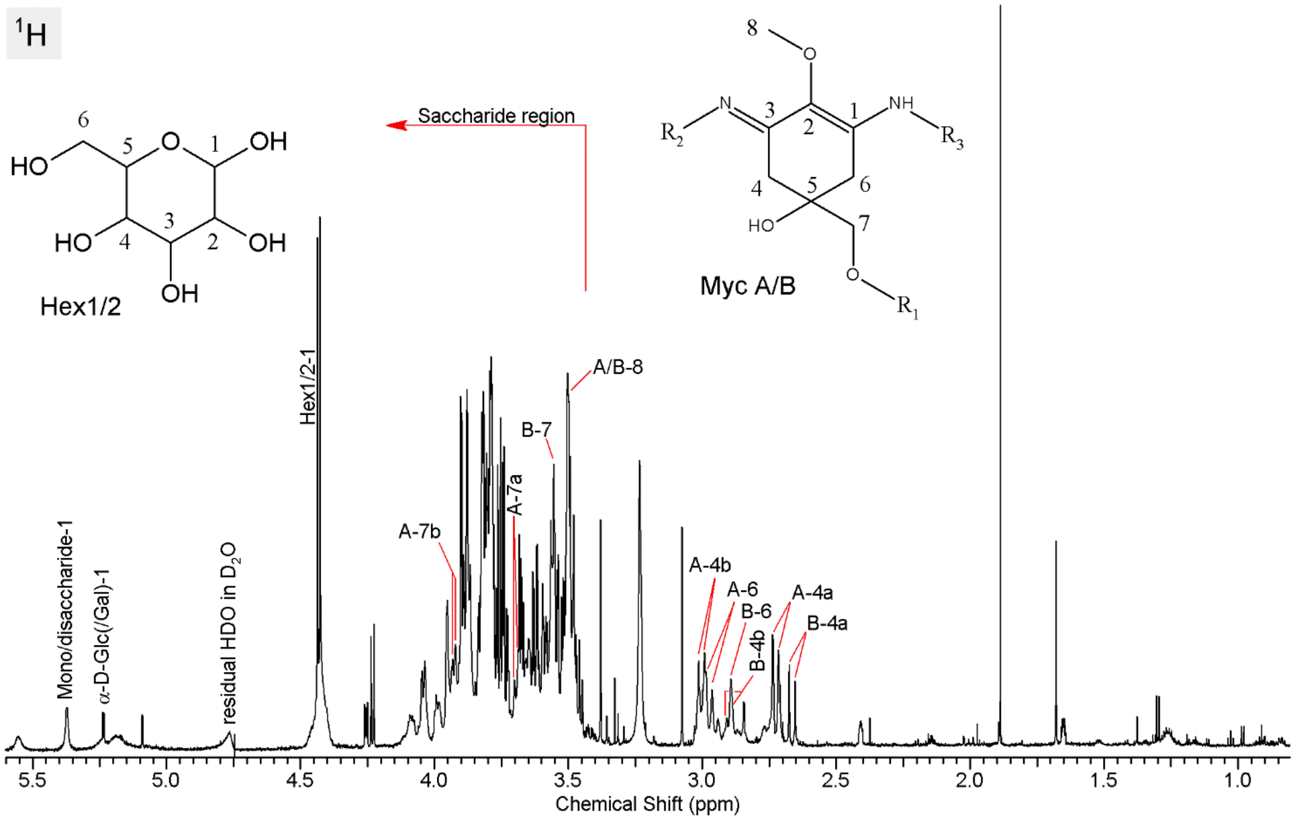

Figure S4. Partly annotated proton spectrum of aplysiapalythine E (**19**) sample in D_2O.

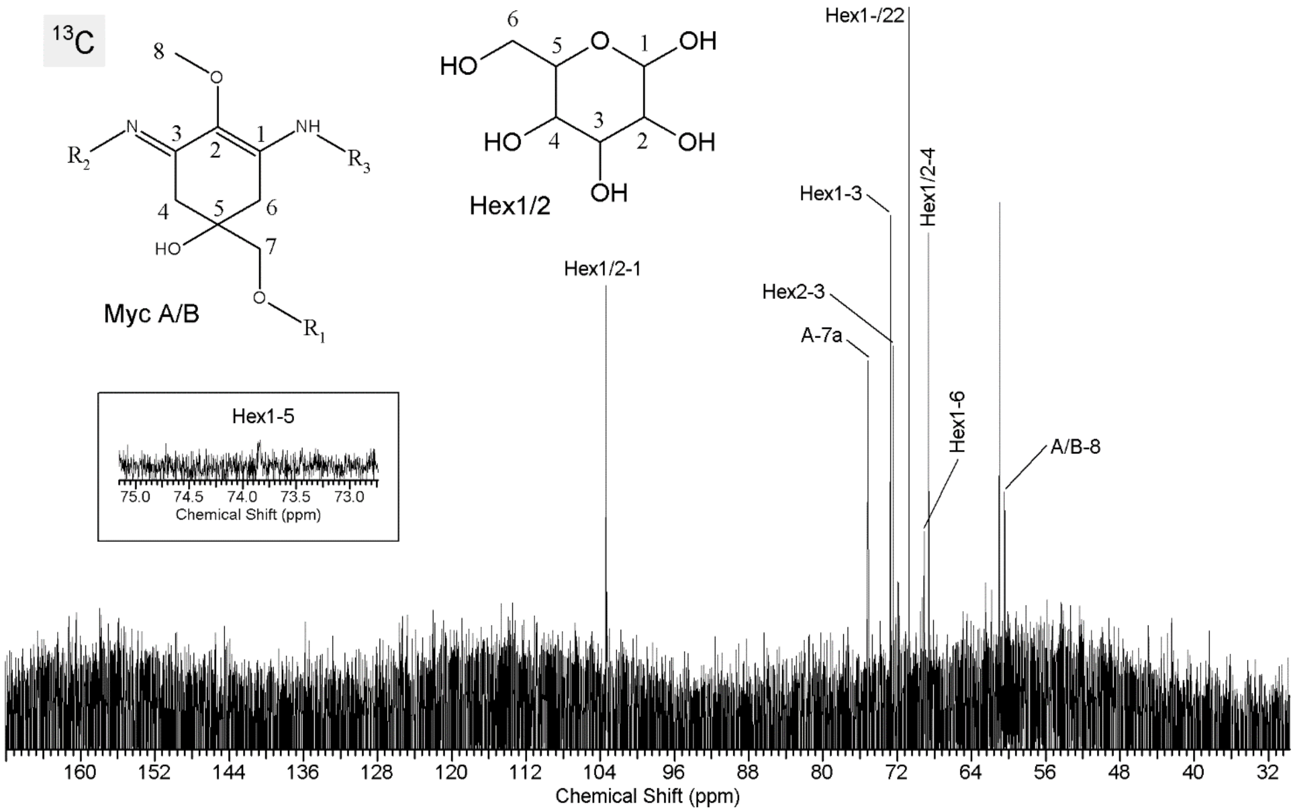

Figure S5. Annotated carbon spectrum of aplysiapalythine E (**19**) sample in D_2O.

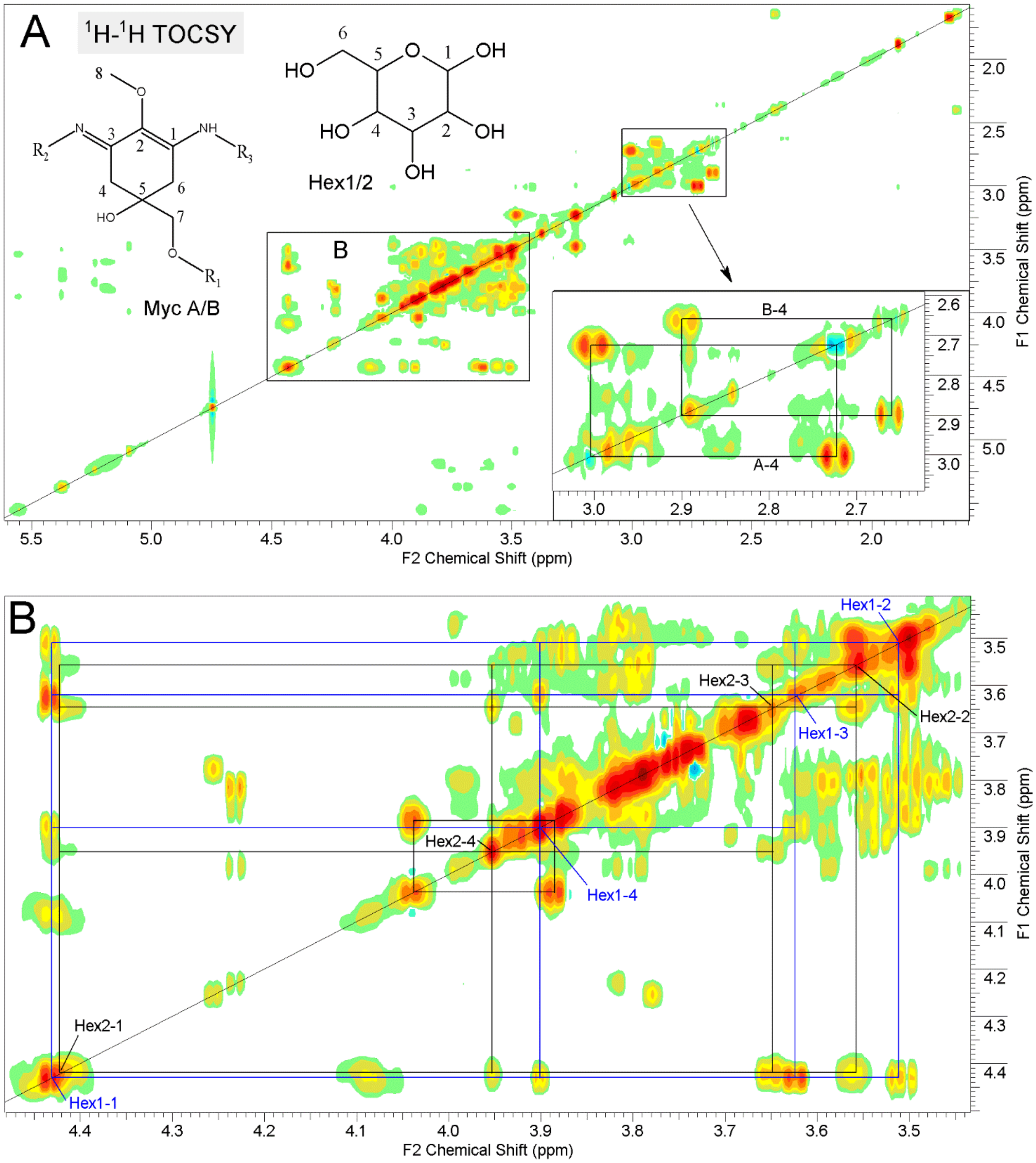

Figure S6. Partly annotated ^{1}H-^{1}H TOCSY (90 ms) spectrum of aplysiapalythine E (**19**) sample in D_2O with enlargement from area B.

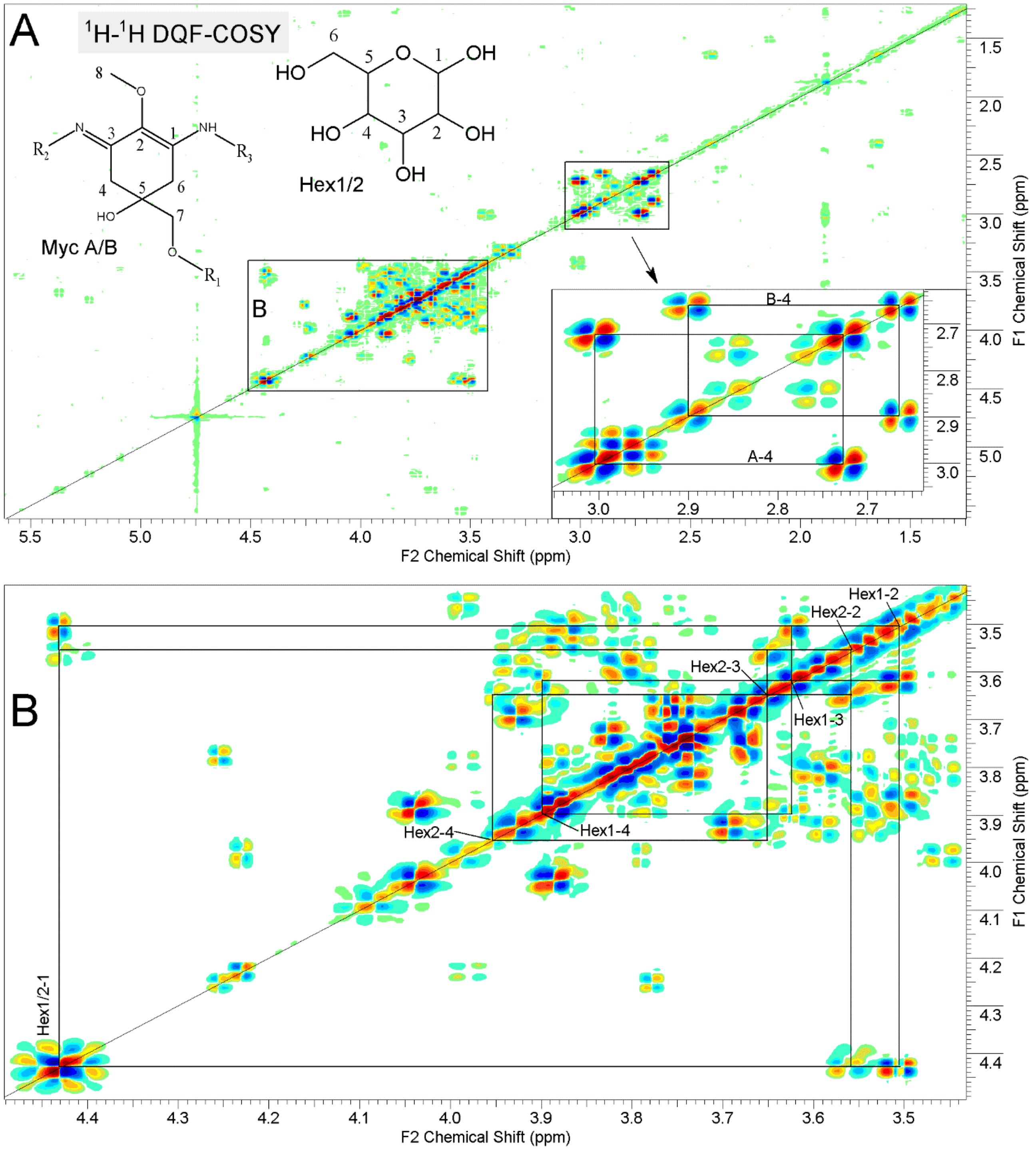

Figure S7. Partly annotated ^{1}H-^{1}H COSY spectrum of aplysiapalythine E (**19**) sample in D_2O with enlargement from area B.

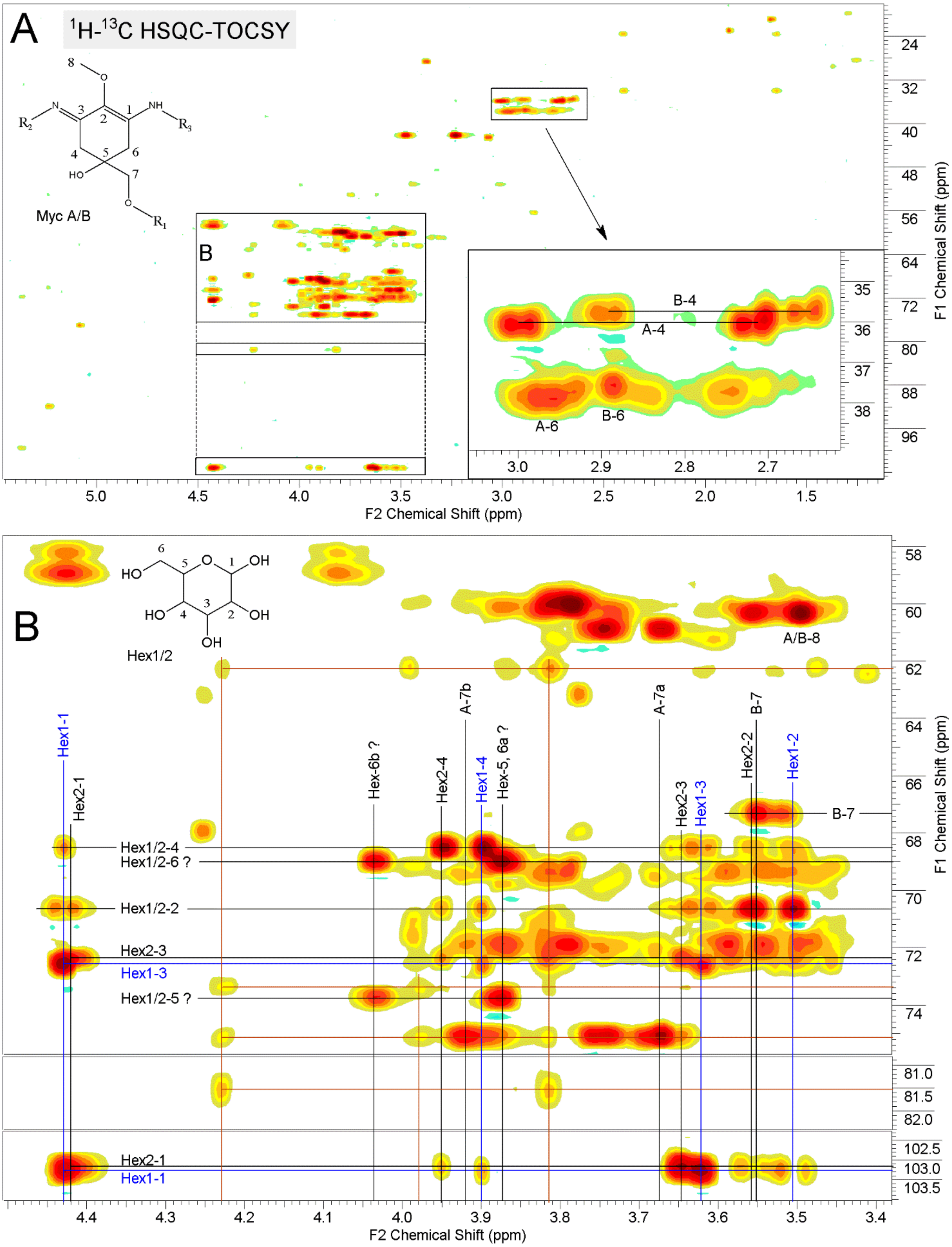

Figure S8. Partly annotated ^{1}H-^{13}C HSQC-TOCSY spectrum of aplysiapalythine E (**19**) sample in D_2O with enlargement from area B.

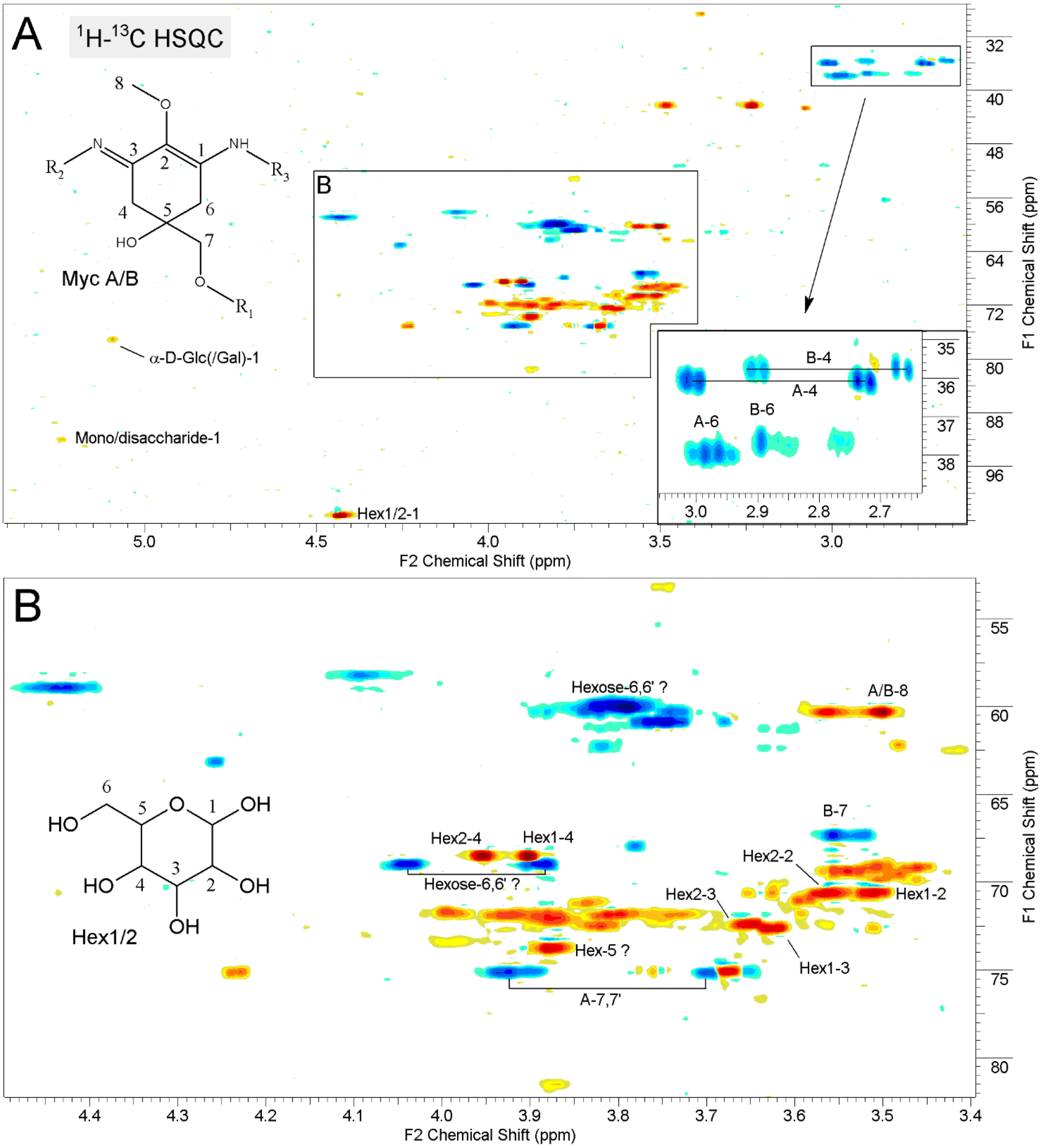

Figure S9. Partly annotated edited ^{1}H-^{13}C HSQC spectrum of aplysiapalythine E (**19**) sample in D_2O with enlargement from area B.

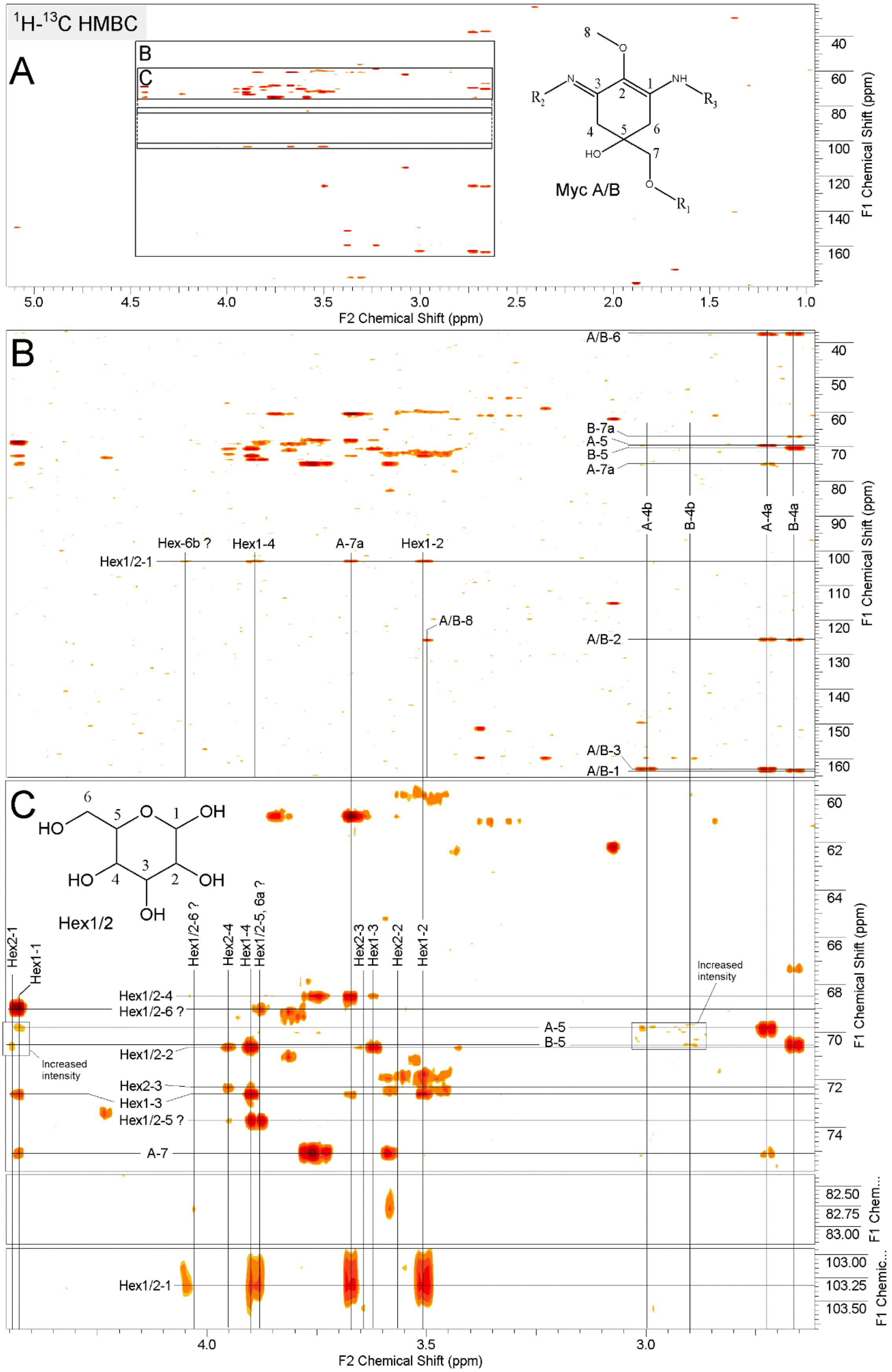

Figure S10. ^{1}H-^{13}C HMBC spectrum of aplysiapalythine E (**19**) sample in D_2O with enlargements from area B and C.

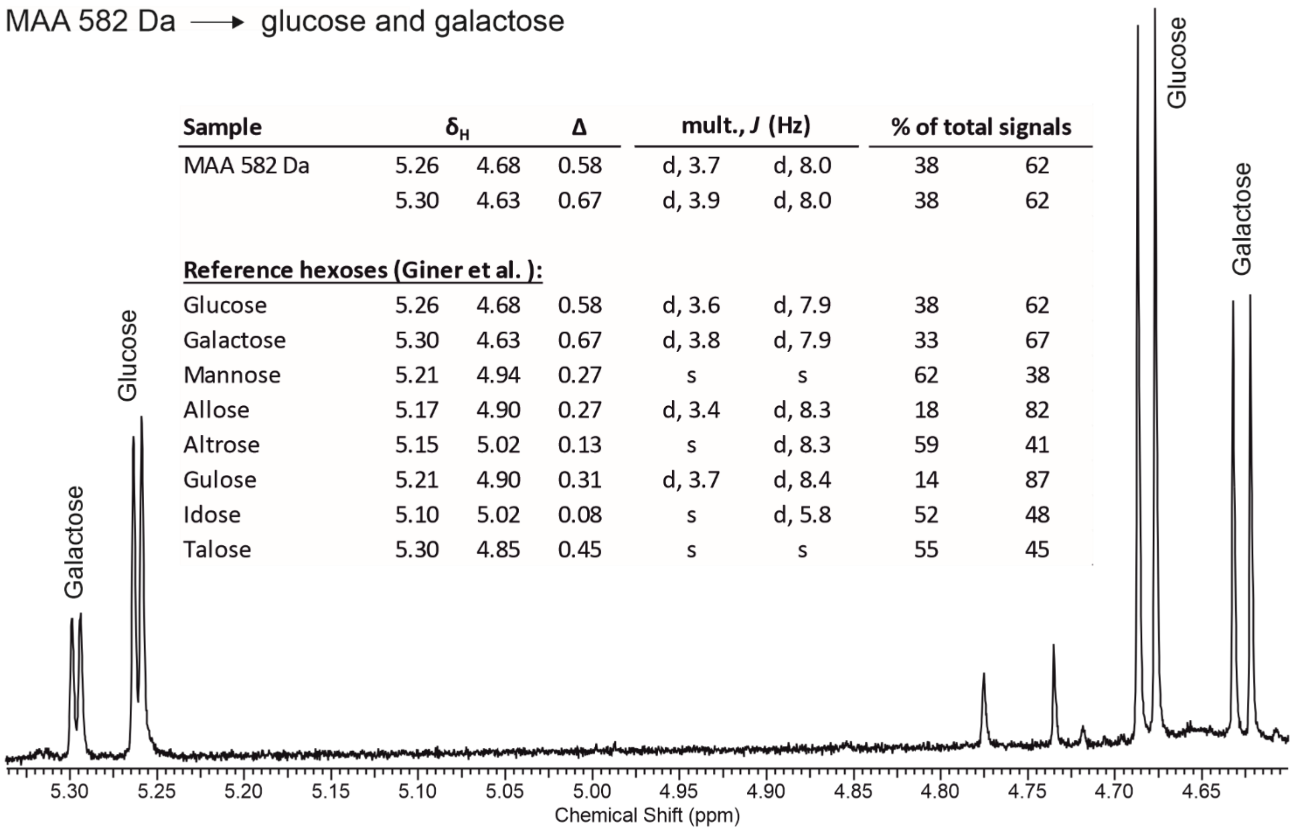

Sample	δ_H		Δ	mult., *J* (Hz)		% of total signals	
MAA 582 Da	5.26	4.68	0.58	d, 3.7	d, 8.0	38	62
	5.30	4.63	0.67	d, 3.9	d, 8.0	38	62
**Reference hexoses (Giner et al.):**							
Glucose	5.26	4.68	0.58	d, 3.6	d, 7.9	38	62
Galactose	5.30	4.63	0.67	d, 3.8	d, 7.9	33	67
Mannose	5.21	4.94	0.27	s	s	62	38
Allose	5.17	4.90	0.27	d, 3.4	d, 8.3	18	82
Altrose	5.15	5.02	0.13	s	d, 8.3	59	41
Gulose	5.21	4.90	0.31	d, 3.7	d, 8.4	14	87
Idose	5.10	5.02	0.08	s	d, 5.8	52	48
Talose	5.30	4.85	0.45	s	s	55	45

Figure S11. Partial ^{1}H spectra of aplysiapalythine E (**19**) acid hydrolysate in 2 M D_2SO_4 (in D_2O) showing the match of anomeric protons signals to reference compounds presented in the table which values are from Giner et al., J Nat Prod 2016, 79, 2413-2417.

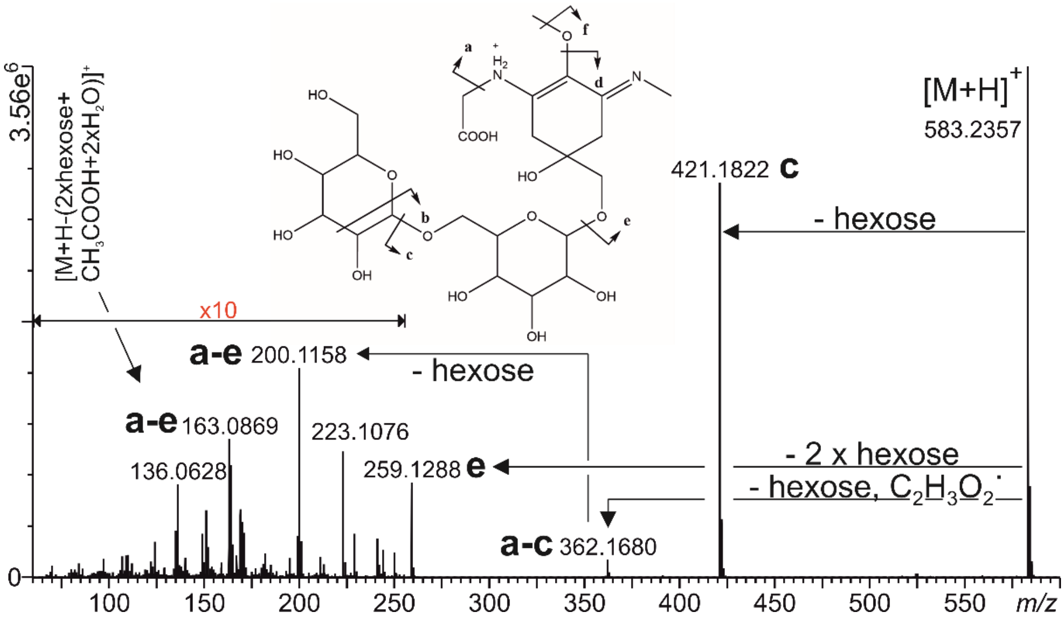

Figure S12. Product ions of aplysiapalythine E (**19**) from MS^E spectrum. Line with arrow ends shows the magnified range of the spectrum and the red x-number the magnification.

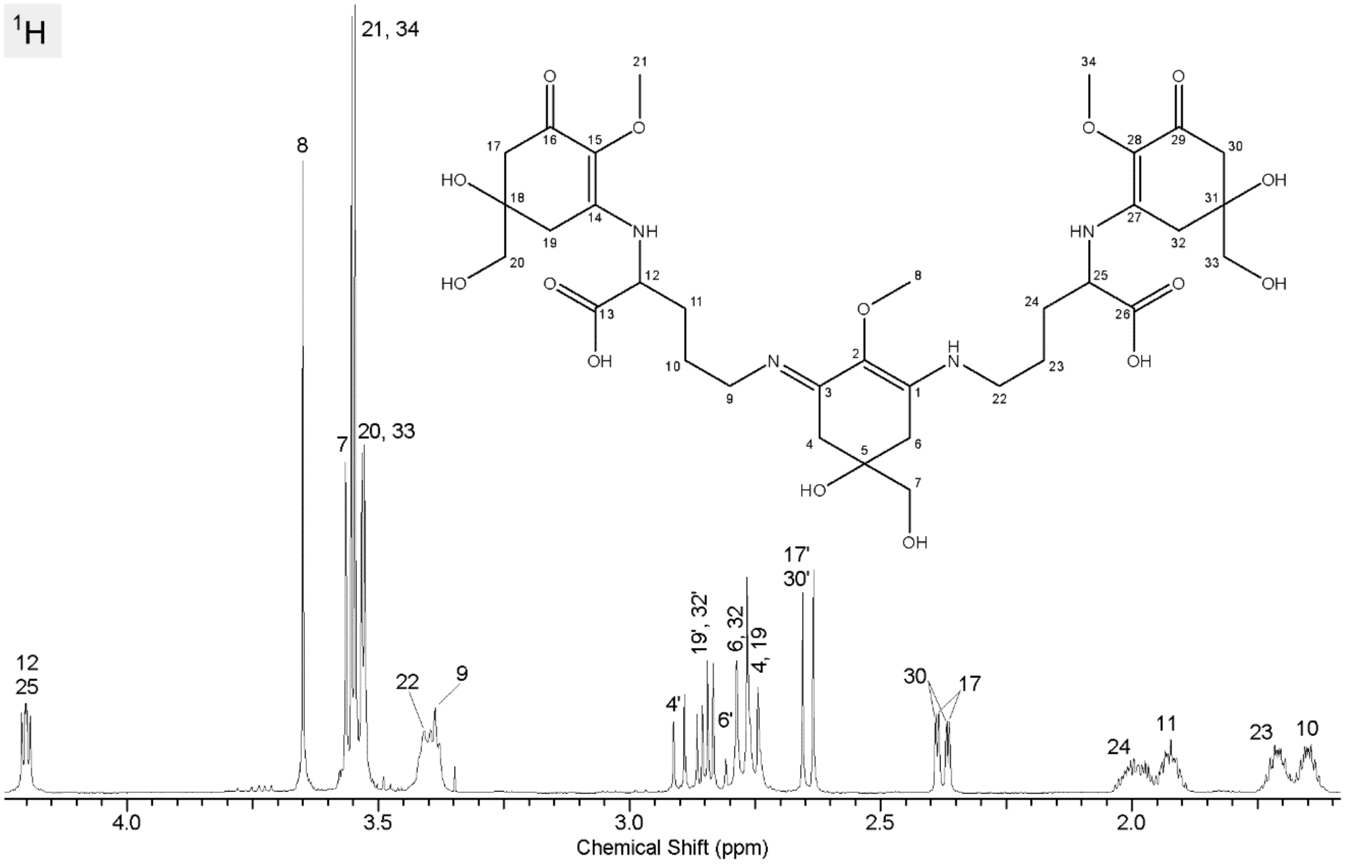

Figure S13. Annotated proton spectrum of tricore B (**18**) in D_2O.

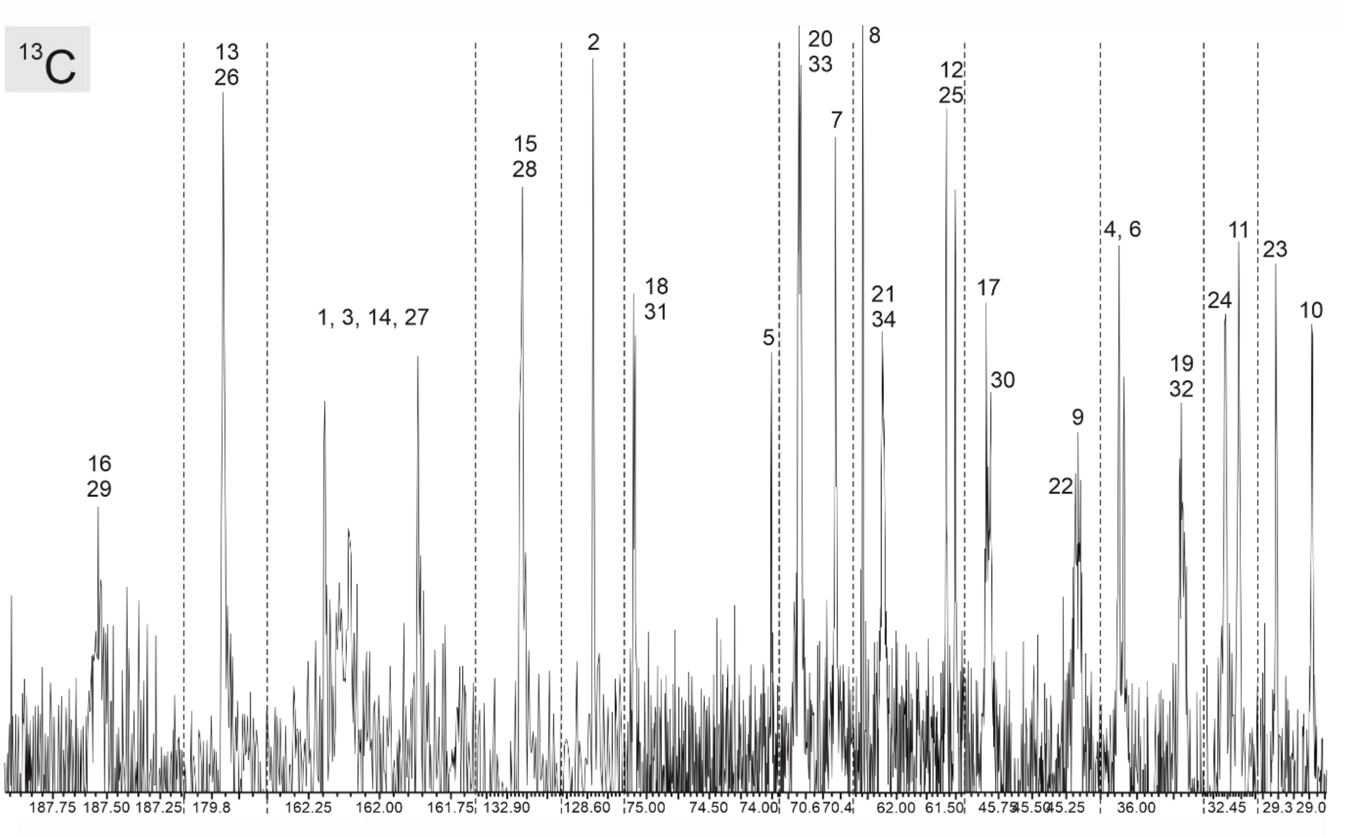

Figure S14. Annotated carbon spectrum of tricore B (**18**) in D_2O.

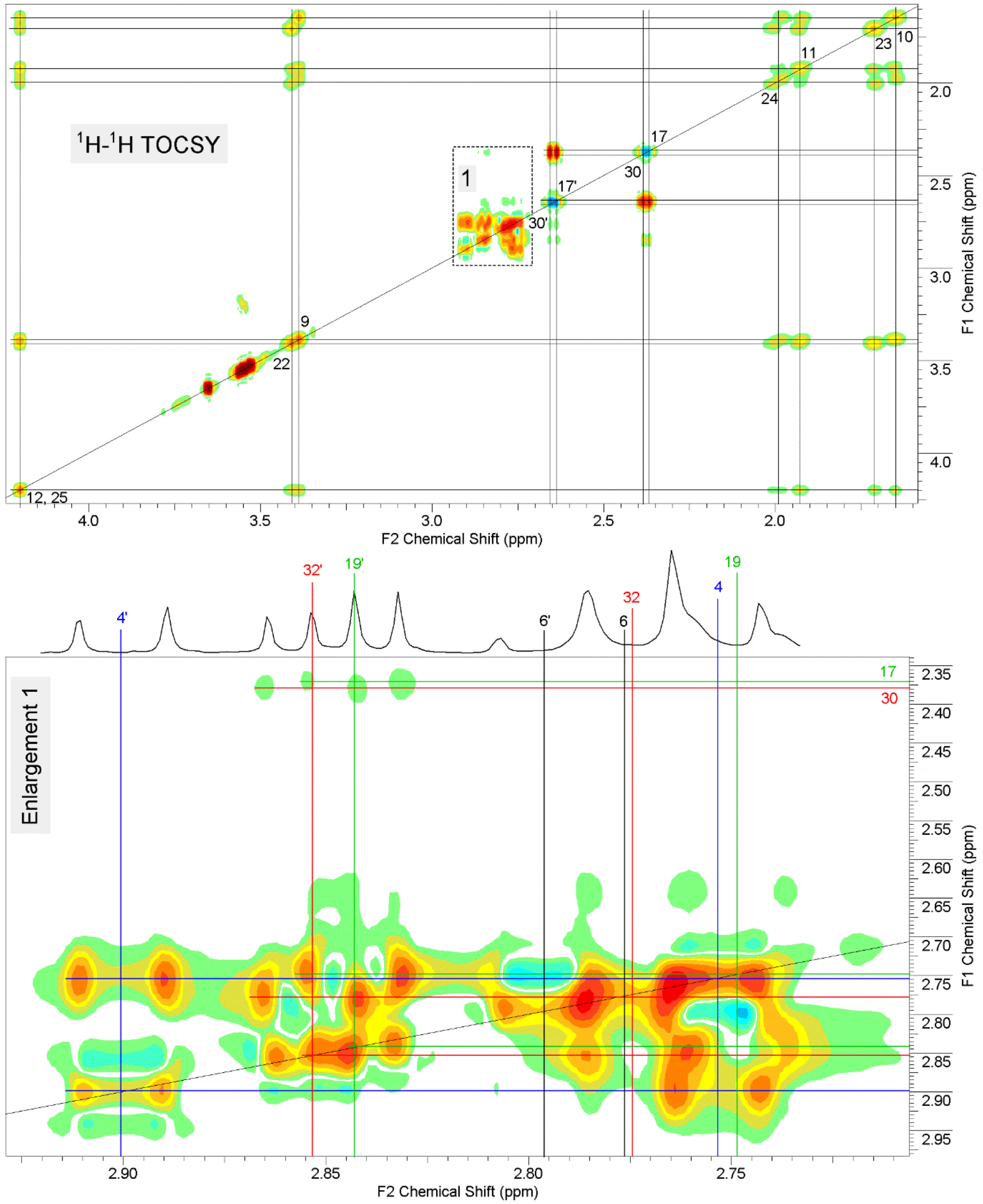

Figure S15. Annotated ^{1}H-^{1}H TOCSY (90 ms) spectrum of tricore B (**18**) in D_2O with enlargement from area. 1.

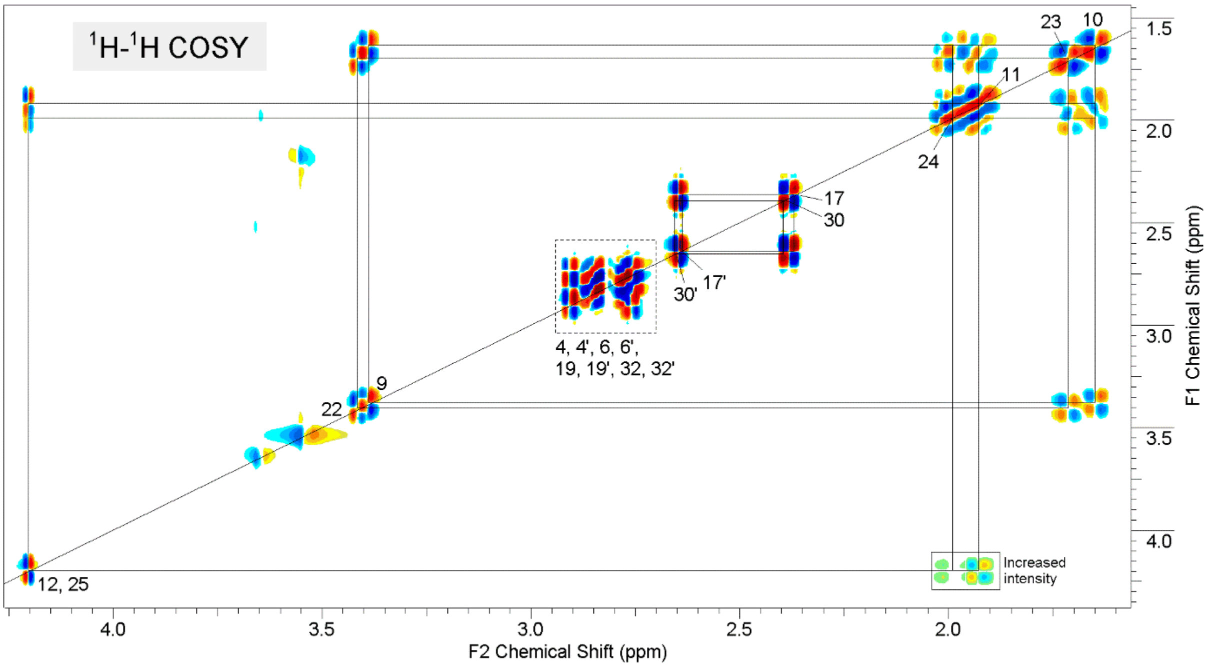

Figure S16. Annotated ^{1}H-^{1}H COSY spectrum of tricore B (**18**) in D_2O.

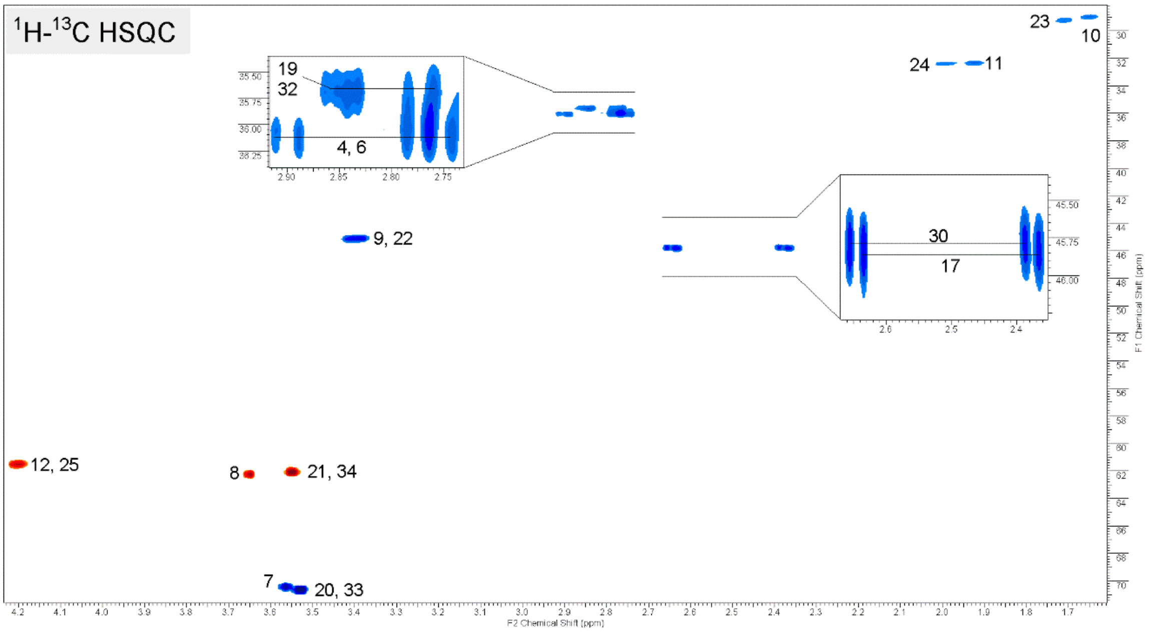

Figure S17. Annotated 1H-13C HSQC spectrum of tricore B (**18**) in D2O.

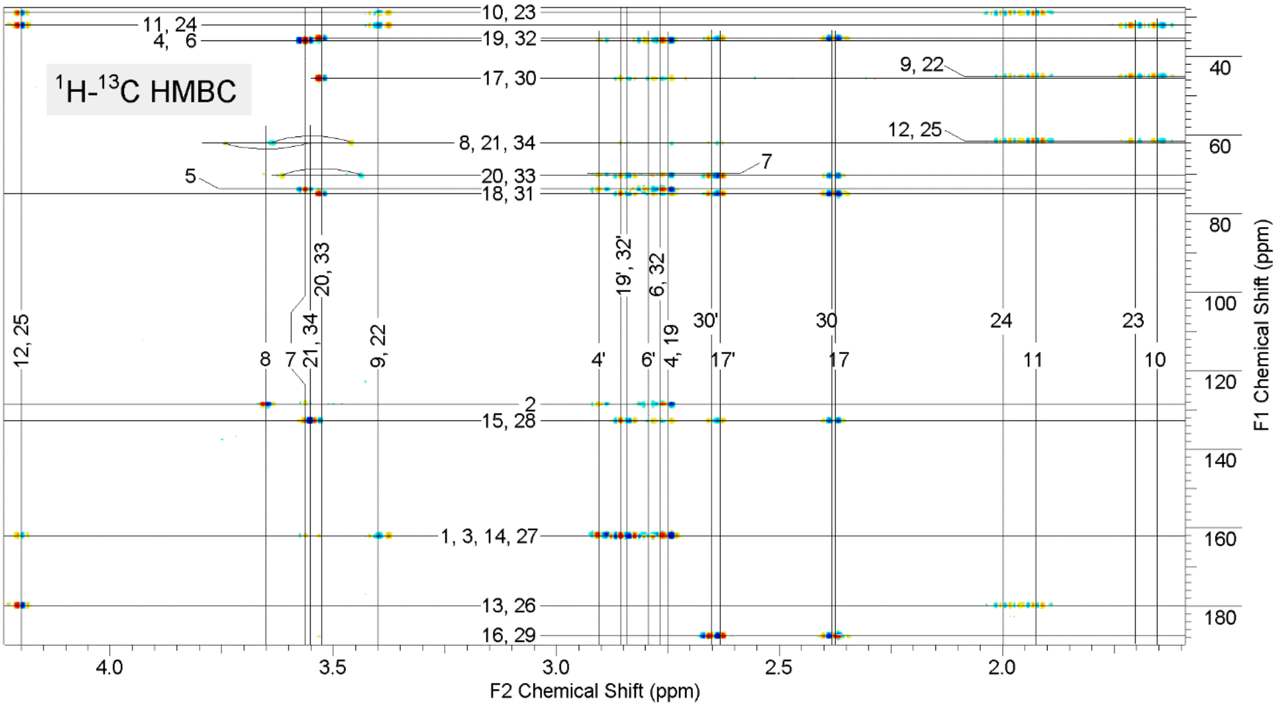

Figure S18. Annotated ^{1}H-^{13}C HMBC spectrum of tricore B (**18**) in D_2O.

Table S5. Size of the chromosomal and plasmid DNA in complete genome assembly of *Nostoc* sp. UHCC 0926.

**DNA Fragment**	**Length**
**Chromosome**	6618685 bp
**Plasmid 1**	1036473 bp
**Plasmid 2**	291576 bp
**Plasmid 3**	218101 bp
**Plasmid 4**	76405 bp
**Plasmid 5**	37024 bp
**Plasmid 6**	35895 bp

Table S6. BlastP hits for the MAA biosynthetic enzymes of *Nostoc* sp. UHCC 0926 based on max score values. (Size based on amino acids. Sequence ID %: Sequence identity percentage)

Gene name	Locus Tag	Size	Sequence ID %	Homolog locus, Origin	Predicted Function
mysA	PQG02_22290	410	95.61	BBB38336.1, *Nostoc commune* KU002	3-dehydroquinate synthase
mysB	PQG02_22295	277	94.95	WP_069074325.1, *Nostoc* sp. KVJ20	Class-I SAM-dependent methyltransferase
mysC₁	PQG02_22305	465	91.83	WP_229462886.1, *Nostoc* sp. CHAB 5824	ATP-grasp domain containing protein
mysD	PQG02_04320	342	92.69	WP_100900872.1, *Nostoc flagelliforme*	D-alanine D-alanine ligase
mysC₂	PQG02_04325	460	92.37	WP_196527594.1, *Nostoc commune*	ATP-grasp domain containing protein
mysC₃	PQG02_04335	442	96.15	WP_229555536.1, *Nostoc* sp. XA010	ATP-grasp domain containing protein
Pseudo mysC₁	PQG02_36110	80	75.95	WP_096682821.1, *Nostoc* sp. NIES-2111	ATP-grasp domain containing protein
mysF	PQG02_36115	350	94.55	MBR8837274.1, *Stigonema ocellatum* SAG 48.90=DSM 106950	Methyltransferase containing-domain protein
mysI	PQG02_36120	363	86.09	MBR8837277.1, *Stigonema ocellatum* SAG 48.90 = DSM 106950	TauD/TfdA family dioxygenase
mysE	PQG02_36125	1143	85.43	MBR8837279.1, *Stigonema ocellatum* SAG 48.90 = DSM 106950	Amino acid adenylation domain-containing protein

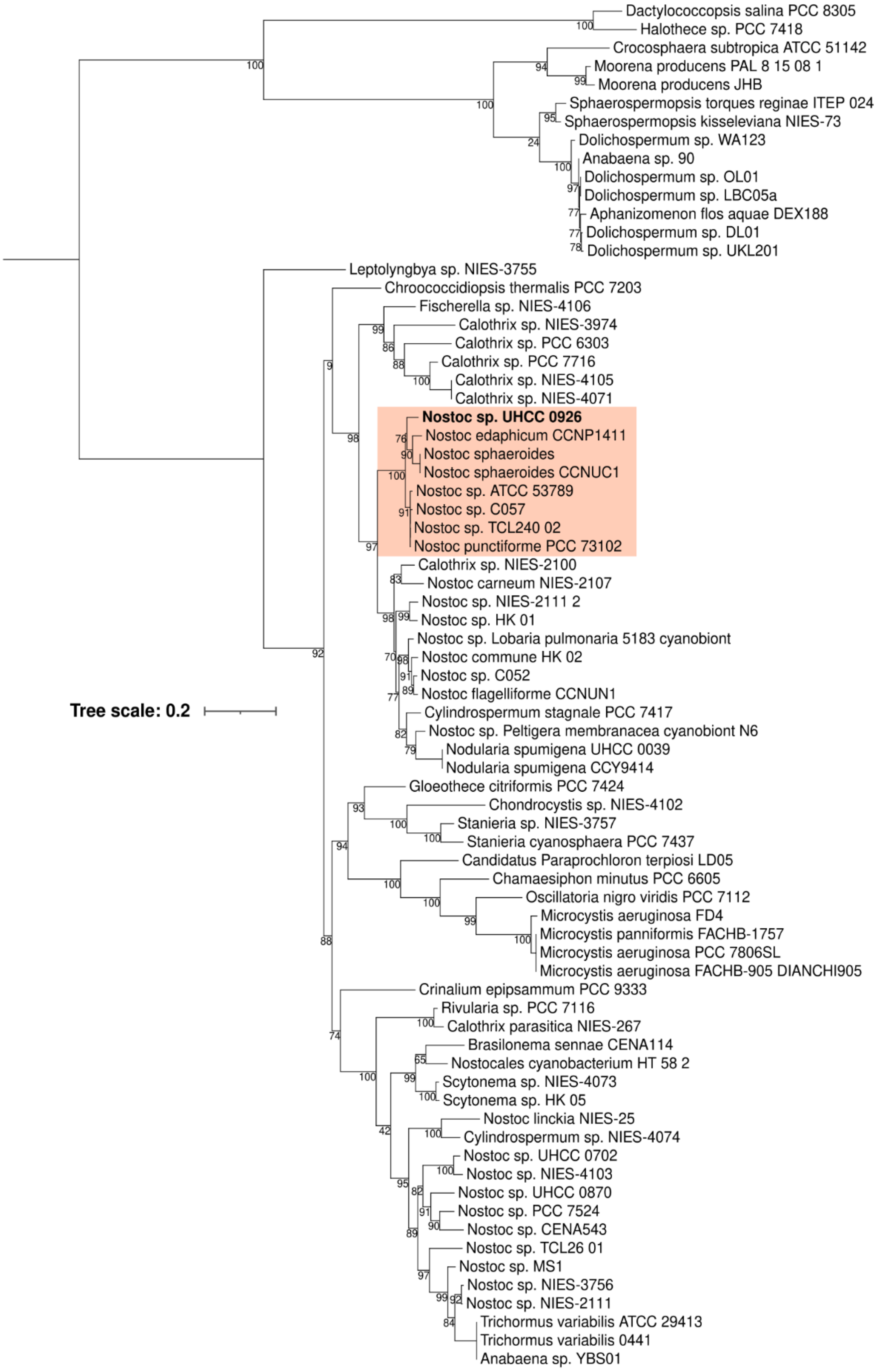

Figure 19. Maximum likelihood phylogenetic tree constructed using MysA amino acid sequences. Clade where the MysA of *Nostoc* sp. UHCC 0926 is placed is highlighted in orange box. Support values are based on 1000 bootstraps.

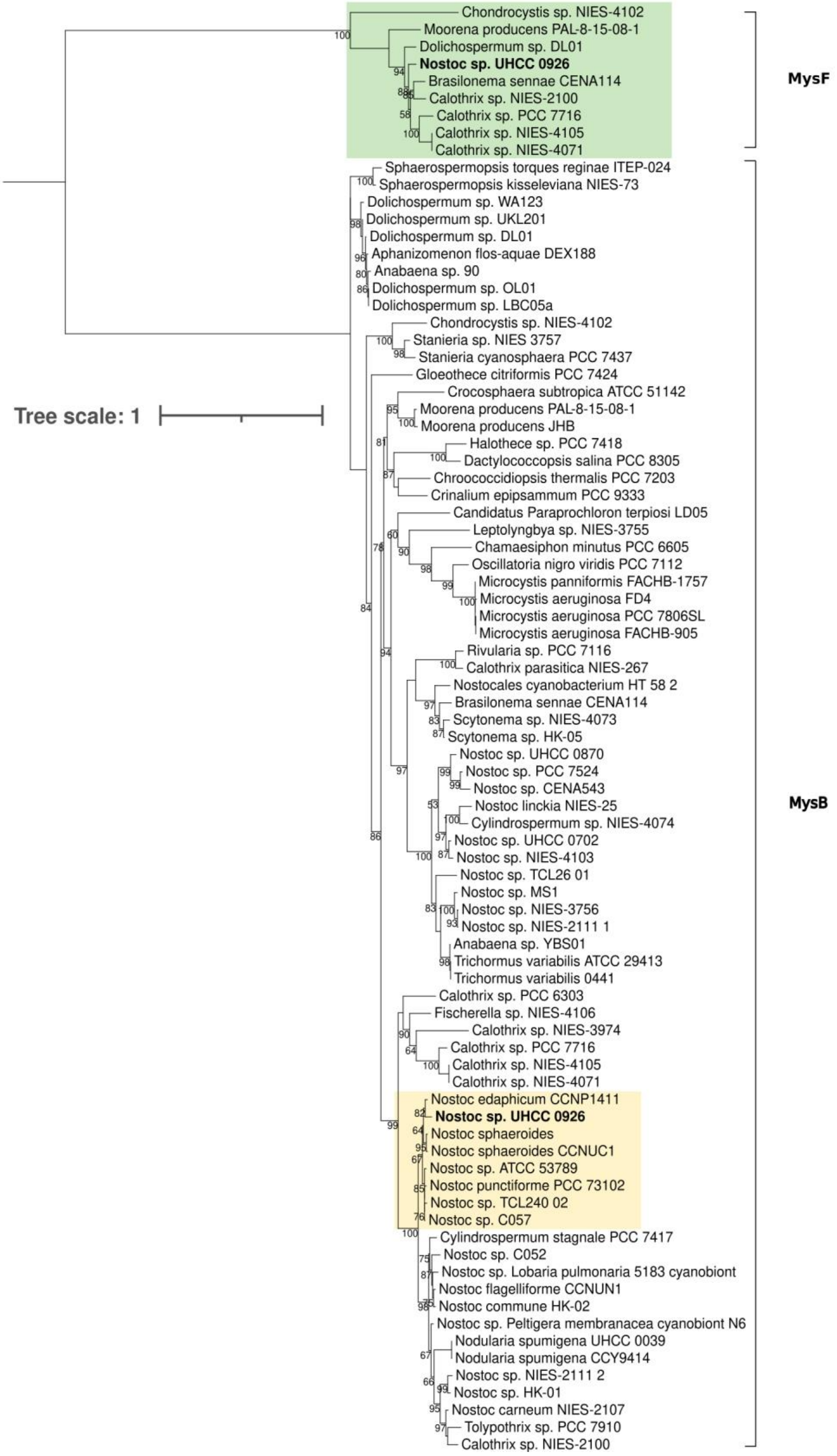

Figure S21: Phylogenetic tree constructed via maximum likelihood method using MysB amino acid sequences along with the additional methyltransferase enzymes identified in the MAA biosynthetic gene clusters. Clade where the MysB of *Nostoc* sp. UHCC 0926 is placed is highlighted in yellow. Support values are based on 1000 bootstraps.

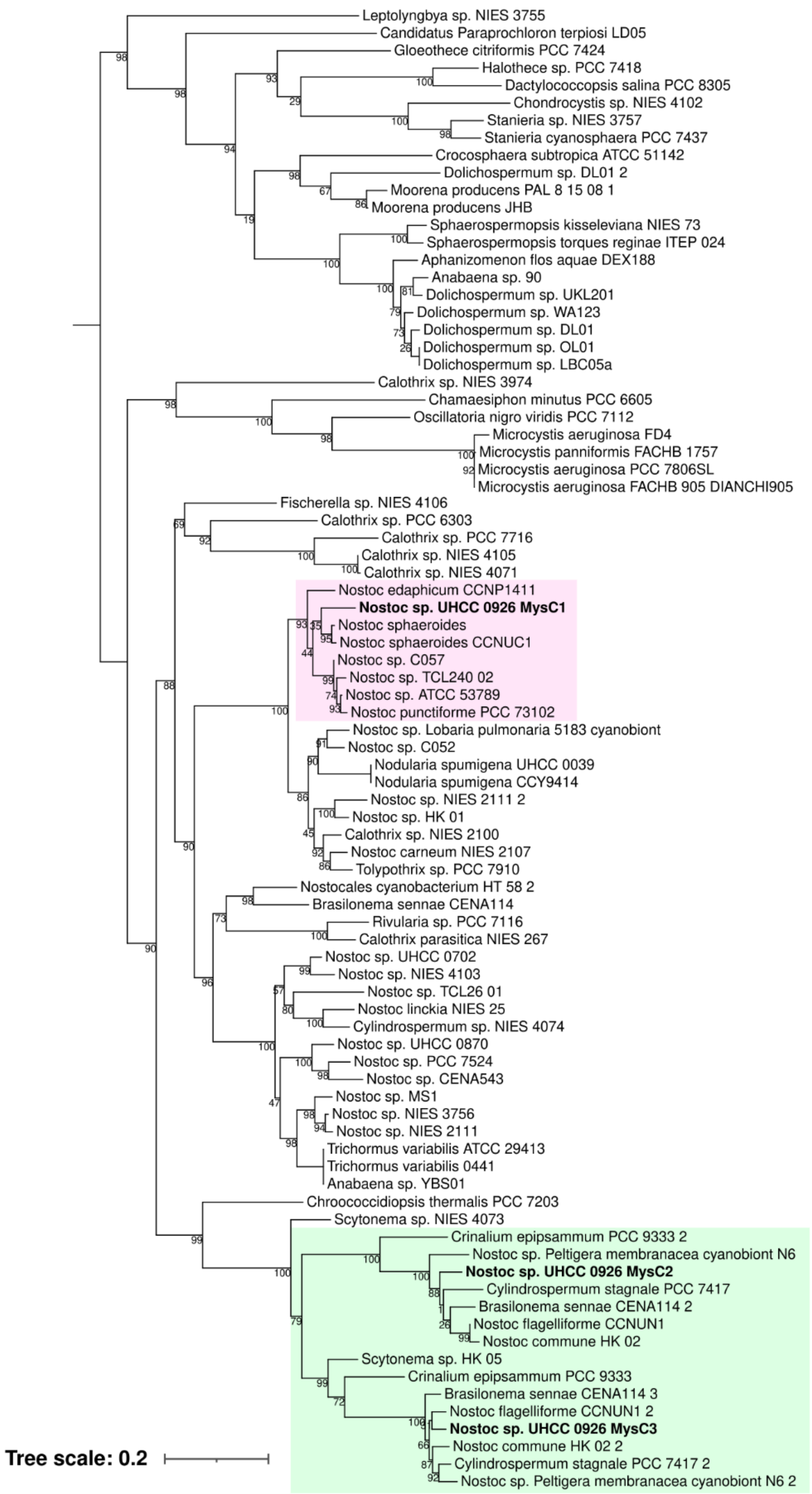

Figure S22: Phylogenetic tree constructed using the MysC amino acid sequences**.** MysC$_1$ (pink) and C$_2$/C$_3$ (green) of *Nostoc* UHCC 0926 are highlighted in their corresponding clades. Support values are based on 1000 bootstraps.

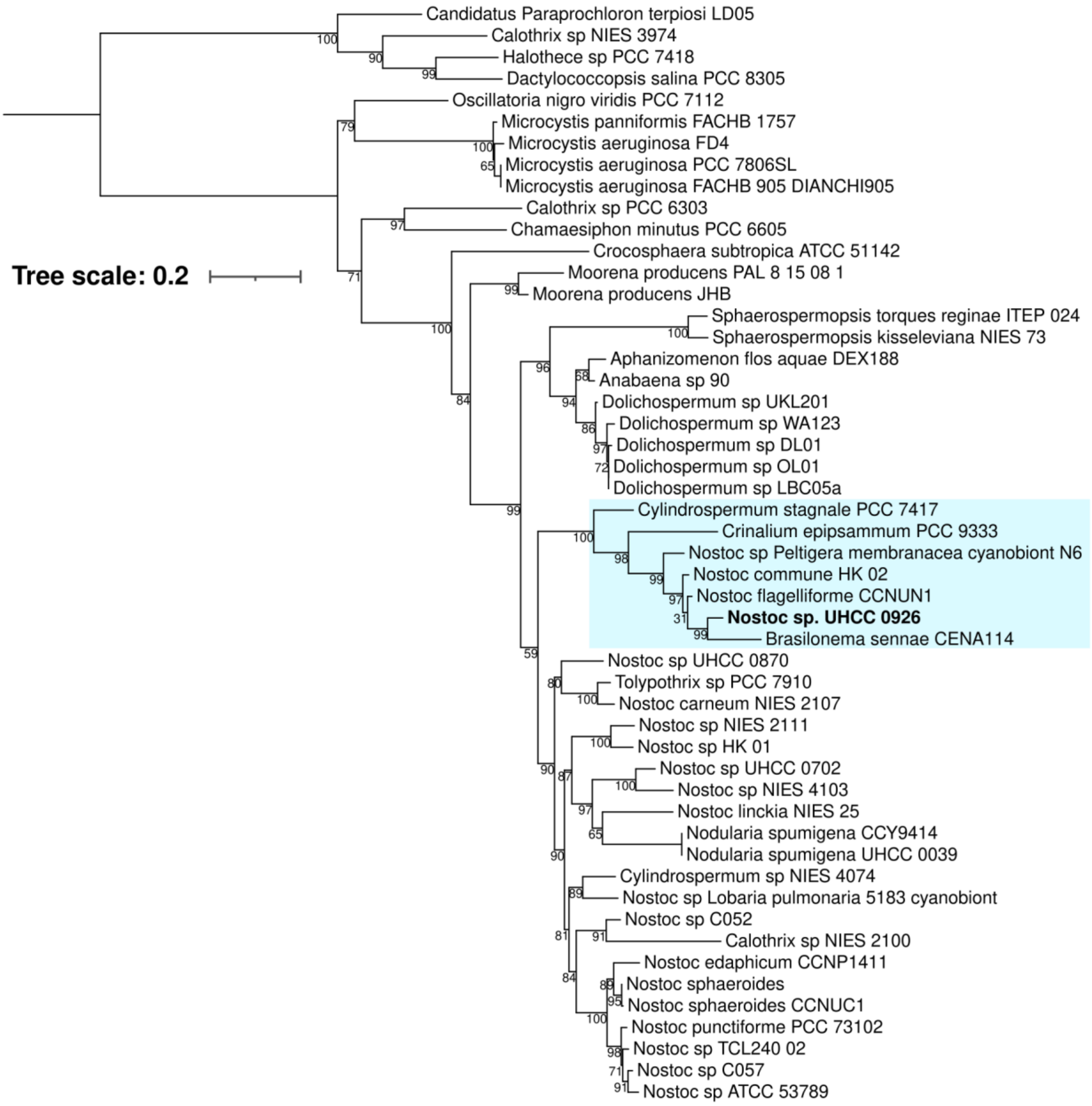

Figure S23: Maximum likelihood phylogenetic tree constructed using MysD amino acid sequences. Clade where the MysD of *Nostoc* sp. UHCC 0926 is placed is highlighted in blue. Support values are based on 1000 bootstraps.

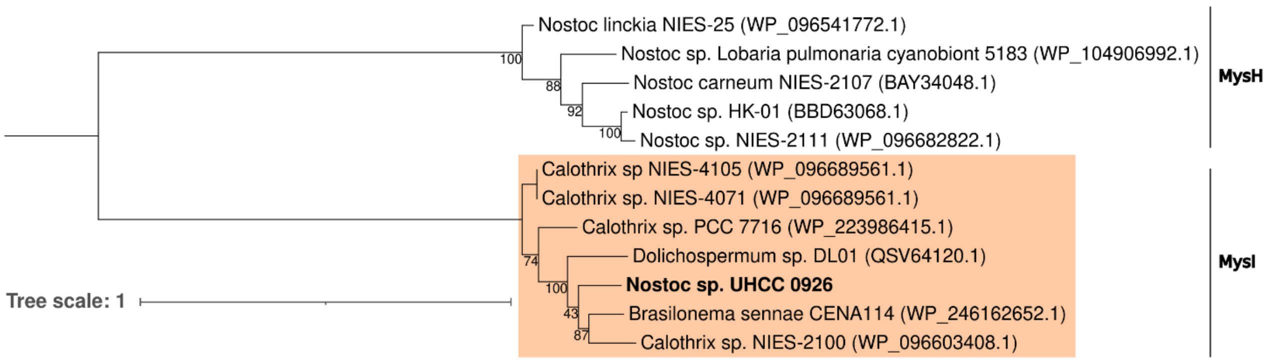

Figure S24. Phylogenetic tree constructed via maximum likelihood method using the alignment of amino acid sequences of MysH enzymes identified within the 10 kb flanking region of the MAA BGC enzymes. Support values are based on 1000 bootstraps.
